# Targeting Tumour Microtubes to Disrupt Glioma Networks

**DOI:** 10.21203/rs.3.rs-8605748/v1

**Published:** 2026-04-07

**Authors:** Tengfei Huang, Po Zhang, Suchet Taori, Shuai Wang, Donghai Wang, Zhiye Chen, Huairui Yuan, Xujia Wu, Tingting Duan, Fanen Yuan, Weichi Wu, Rui Wang, Huan Li, Hailong Mi, Deguan Lv, Deobrat Dixit, Frank Winkler, Qiulian Wu, Jeremy N. Rich

**Affiliations:** 1 Lineberger Comprehensive Cancer Center, University of North Carolina, Chapel Hill, NC, USA; 2 UPMC Hillman Cancer Center, Pittsburgh, PA, USA; 3 Department of Medicine, University of Pittsburgh, Pittsburgh, PA, USA; 4 Herbert Irving Comprehensive Cancer Center, Columbia University Medical Center, New York, NY 10032, USA; 5 Clinical Cooperation Unit Neurooncology, German Cancer Consortium (DKTK), German Cancer Research Center (DKFZ), Heidelberg, Germany; 6 Department of Neurology, University of Pittsburgh, Pittsburgh, PA, USA; 7 Department of Neurology, University of North Carolina, Chapel Hill, NC, USA; 8 Lead contact and to whom correspondence should be addressed

**Keywords:** glioblastoma, glioma stem cells, local protein synthesis, tumor microtubes, tumor network, cancer neuroscience

## Abstract

Glioma cells form multicellular communication networks through tumour microtubes (TMs), integrating tumour–tumour and neuron–tumour connectivity to sustain growth and therapy resistance. Underlying molecular regulation of TMs and potential targeting strategies have proven elusive. Here, we demonstrate that glioma stem cells (GSCs) preferentially grow TMs, which locally synthesize neurotransmitter receptors and metabolic enzymes to support network communication. Coordinated proteomics and functional screening of TMs identified inner mitochondrial component, FASTKD2, as essential to local protein synthesis. Targeting FASTKD2 attenuates tumour stemness and growth, disrupting coordinated mitochondrial RNA metabolism in TMs, which sustains intercellular communication and tumour proliferation. Structure-function screening revealed antibiotic linezolid inhibited FASTKD2 interactions with mitochondrial RNA, thereby disrupting tumour network communication and augmenting efficacy of therapies targeting neuronal stimulation of tumour cells. Collectively, tumour cells coopt features of neuronal cell biology, including localized protein synthesis, to reinforce TM-mediated glioma network communication, generating therapeutic vulnerabilities.

## Graphical Abstract

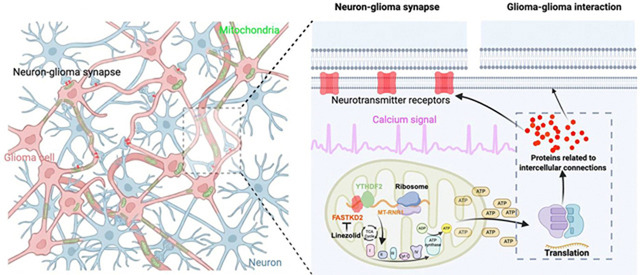

## INTRODUCTION

Neurons promote cancer growth through multiple cellular mechanisms, including direct communication to cancer cells through paracrine and electrochemical mechanisms. Neural activity also regulates the tumour microenvironment. This interplay is inherently bidirectional: tumours remodel neural circuits in both the central and peripheral nervous systems to establish malignant feedback loops, while neurons structurally integrate into the tumour architecture, facilitating communication even over long distances.^1,2^ Glioma cells form glutamatergic synapses with neurons to promote tumour growth^3,4^ and invasion.^5^ Neuron-to-glioma synapse unidirectionally with neurons presynaptic to glioma cells to induce excitatory postsynaptic currents (EPSCs), predominately mediated by calcium-permeable AMPA (alpha-amino-3-hydroxy-5-methyl-4-isoxazolepropionic acid) receptors (AMPAR) on glioma cells^3,4^ with GABAergic neurons important in diffuse midline gliomas^6^. EPSCs depolarize tumour cells and stimulate proliferation, and pharmacological inhibitors of signal transmission attenuate glioma cell proliferation and invasion.^3–5^

Gliomas form intercellular networks with other tumour cells and with neurons. Networks are generated through the formation of ultralong, neurite-like membrane protrusions designated tumour microtubes (TMs), which promote tumour cell proliferation^7^ and invasion into brain.^5–8^ Glioma TMs express neurotransmitter receptors and synaptic signalling molecules, communicating with the tumour microenvironment^4,9,10^. Increased neuronal activity through neuron-to-glioma connections promotes tumour growth and invasion through the release of activity-regulated mitogenic paracrine factors and electrochemical calcium-mediated gap junctions.^4,11–13^ TMs also enable multicellular communication through intercellular Ca^2+^ waves.^7,8,14^ Glioma networks display self-repair capacity after surgical disruption and enhance resistance to conventional chemotherapy (temozolomide, TMZ) and radiotherapy^15,16^. TMs support movement of subcellular materials and organelles, such as nucleic acids and mitochondria, thereby promoting tumour maintenance and proliferation^17–19^. Astrocytes and glioma TMs horizontally transfer mitochondria in GBM^18^, potentially via tunnelling nanotubes (TNTs). Multicellular networks and TMs are detectable in diverse categories of human gliomas, including IDH-wildtype glioblastomas (GBMs), IDH mutant-gliomas, and K27M-mutated midline gliomas, in association with therapeutic resistance, suggesting broad contributions to tumour biology.^3,4,7– 8,20 -21^

Leveraging these connections between cancer and neuroscience to translate into cancer therapies has focused attention on the molecular underpinnings driving the formation and maintenance of multicellular networks. Pharmacological AMPAR inhibition with the anti-epileptic drug perampanel disrupts glioma cell proliferation and invasion.^3–5^ Less well understood is the molecular governance of the formation and maintenance of TMs and their connections. The membrane neuronal developmental regulator, tweety-homolog 1 (Ttyh1), promotes TM-mediated brain invasion by glioma cells, and targeting Ttyh1 inhibited the formation of invasive TMs.^7,8^ Connections between TMs and synapsed cells are mediated through gap junctions (prominently connexin 43) and adherens junctions to permit intercellular communication through calcium waves and exchange of small molecules, phenocopying astrocyte networks.^3,7,22^ Preclinical efficacy by targeting gap junction function against gliomas was reported prior to discovery of TMs and network functions, including in augmentation of efficacy of cytotoxic therapies, like radiation and chemotherapy.^3,15,23^ With the recognition of multicellular networks, the role of intercellular communication has become heightened as glioma cells lacking connections lack resilience and respond to cytotoxic therapies.^3 ,21,15,24^

Brain tumours have been one of the hallmark cancers in which intratumoural cellular diversity resembles aspects of neural development with a self-renewing, highly tumourigenic stem-like tumour cell population, designated as cancer stem cells, at the apex of a cellular hierarchy.^25–27^ Like networked tumour cells, cancer stem cells display high resilience and therapeutic resistance with active interplay with the tumour microenvironment.^10,28^ GSCs share some molecular regulators of neuronal crosstalk to tumour cells, e.g. BDNF (brain-derived neurotrophic factor), which promote GSC maintenance and tumour growth.^29–30^ Connections between neuronal activity and stem cell biology have been established in normal development and tissues homeostasis as tissue stem cell niches receive innervation that regulates including in the skin,^31–33,^ gastrointestinal tract,^34^ and bone marrow.^35^ Thus, there is a convergence between cancer neuroscience and cancer stem cells. Indeed, the presence of TMs has been hypothesized to promote aspects of cancer stemness.

Despite an emerging understanding of glioma cell connectivity to other cells in the tumour microenvironment, much remains to be uncovered regarding the specific molecular mechanisms underlying the establishment, functionality, role, and therapeutic vulnerability of GSC TMs. Here, we demonstrate that GSCs utilize local protein synthesis in TMs to promote communication and survival in the tumour microenvironment. Pharmacologically targeting local protein synthesis pathway suppresses glioma growth and offers a therapeutic paradigm for treating brain tumours.

## RESULTS

### Glioma network communication depends on protein synthesis within TMs

**Figure 1.**
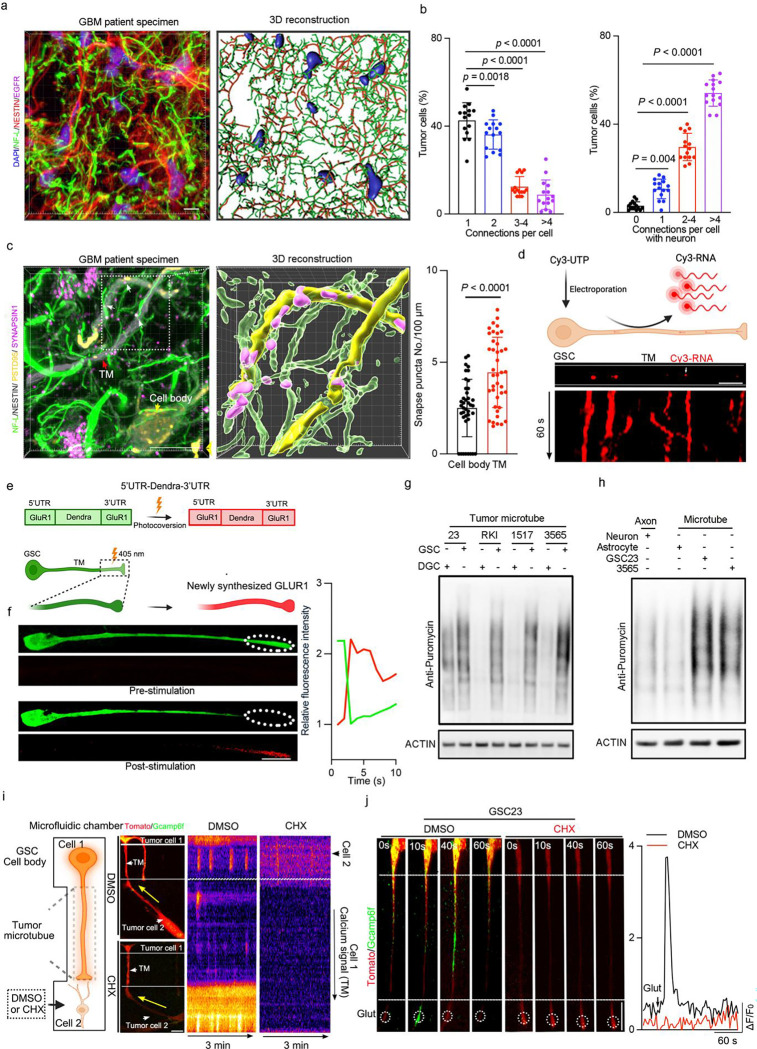
Intercellular communication in glioblastoma depends on local protein synthesis in GSC TMs a Patient-derived glioblastoma surgical biopsy specimens underwent immunofluorescent staining for a tumour cell marker [EGFR (Magenta)], stem cell marker [Nestin (red)], neuron marker [NF-L (Green)], and general marker of nuclei [DAPI (Blue)]. Representative images are shown. Scale bar: 40 μm. Right, three-dimensional reconstruction of tumour cell–tumour cell and tumour cell–neuron networks. b Quantification of tumour–tumour and tumour–neuron interactions. N=3 fields per sample from N=5 patient specimens. c Patient-derived glioblastoma surgical biopsy specimens underwent immunofluorescent staining for presynaptic marker [synapsin1 (Magenta)], stem cell marker [Nestin (red)], neuronal marker [NF-L (Green)], and post synaptic marker [PSD95 (Yellow)]. Arrows indicate synapse puncta. Labelled red arrows indicate TMs and yellow arrows cell bodies. Representative images are shown. Scale bar: 40 μm. Middle, three-dimensional reconstruction of tumour–neuron interaction. Right, quantitative analysis of synapse between glioma and neuron. N = 5 tumour specimens, n=40 cells. d Top: schematic showing labelling of endogenous RNAs in GSC TMs. Bottom: GSC TMs with Cy3-labeled RNA granules moving along the TMs; representative granule dynamics are shown by kymograph analysis. Scale bar, 10 μm. e Graphical schematic depicting the construction of an activatable fluorescent reporter of protein translation using green fluorescence at baseline and red signifying active translation between the 5’ and 3’ untranslated regions (UTRs) of glutamate receptor 1 (GluR1), designated as 5’UTR_GluR1_-Dendra2–3’UTR_GluR1_. f Top left, Graphical representation of experimental design in which patient-derived GSCs were transduced with the 5’UTR_GluR1_-Dendra2–3’UTR_GluR1_ reporter, then allowed to form TMs, which were then locally stimulated through laser light to measure spatial protein translation of GLUR1 through transition from green-to-red. Bottom left, GSC23 cells transfected with 5’UTR_GluR1_-Dendra2–3’UTR_GluR1_, then were locally stimulated at the tips of TMs with laser light. Representative images show localized GluR1 translation at the terminals of TMs. Right, Time course of fluorescence changes after laser stimulation of GSC TMs transfected with the 5’UTR_GluR1_-Dendra2–3’UTR_GluR1_ reporters. g Matched GSCs and DGCs were cultured separately in parallel, then treated with OP-puromycin. TMs were selectively harvested, resolved by SDS-PAGE, and immunoblotted for puromycin to quantify protein synthesis. ACTIN was used as a loading control. h Neurons, astrocytes, or GSCs were cultured separately in parallel, then treated with OP-puromycin. TMs were selectively harvested, resolved by SDS-PAGE, and immunoblotted for puromycin to quantify protein synthesis. ACTIN was used as a loading control. i Experimental design and quantification of calcium wave propagation in microfluidic chambers. Left: Schematic of the experimental setup. GSC23 cells transduced with a dual reporter were cultured in microfluidic chambers to spatially isolate TMs from cell bodies. TMs were locally pretreated with vehicle (DMSO) or CHX (10 μg/ml) for 6 h, followed by co-culture with partner tumour cells (Cell 2) for 24 h. Middle: Representative fluorescence images of the microfluidic co-culture system. Long yellow arrows indicate the direction of calcium signal propagation (from Cell 2 to Cell 1), and short arrows mark individual cells: Tumour Cell 2 (calcium signal donor) and Tumour Cell 1 (calcium signal acceptor). Right: Representative kymographs illustrating calcium dynamics across the TM connection. Scale bar: 30 μm. j Experimental design in which GSCs (GSC23) were transduced with a dual reporter with constitutive expression of dTomato fluorescence reporter and GCaMP6f calcium reporter, then cultured in microfluidic chambers to permit spatial isolation of TMs from cell bodies with vehicle control (DMSO) or local CHX (10 μg/ml) for 6 hours. TMs were given a local glutamate puff (100 μM) and fluorescence imaging was performed over 60 second time courses. Middle: Representative images from (j) are shown. Green (GCaMP6f) denotes glioma calcium signalling, and red (dTomato) provides constitutive imaging. Scale bar: 40 μm. Right: Quantification of calcium levels measured by GCaMP6f intensity for GSC responses to glutamate stimulation with local DMSO or CHX pretreatment. Representative of three independent experiments in d, f, g, h. Data are presented from three independent experiments in d, f, g, h. Data are presented as mean ± SD. Two-tailed paired Student’s t-test with p values for c. One-way ANOVA followed by multiple comparisons with adjusted p-values for b.

Glioma TMs provide structures mediating intercellular connectivity and signal transmission. Given GSCs central role in coordinating tumour growth, we hypothesized that GSCs preferentially form TMs and engage both in neuron–tumour communication and tumour cell-tumour cell communication. To address this, we performed high-resolution 3D reconstruction and topological quantification of human GBM surgical specimens stained with GSC markers (SOX2 and NESTIN), revealing highly interconnected networks involving GSCs as hubs with a high connectivity indices. These cells extended neurite-like tumour microtubes (~100 μm), consistent with TMs, which physically interfaced with neuronal elements (assessed by NF-L staining) and neighbouring tumour cells (Fig. 1a–c, Extended Data Fig. 1a). TMs serve as specialized signalling conduits enriched with synaptic machinery, which we confirmed by accumulation of a synaptic marker (PSD95) and AMPAR subunits (GluR1/2) along the protrusions relative to cell somas (Fig. 1c, Extended Data Fig. 1b). GAP43, which is associated with TM formation, was more highly expressed in GSCs than in either neural stem cells (NSCs) or differentiated glioma cells (DGCs) (Extended Data Fig. 1c). In silico analysis of data from The Cancer Genome Atlas (TCGA) revealed that GSCs exhibited elevated expression of neural development–related gene signatures compared to DGCs (Extended Data Fig. 1d), and expression of neuron and axon-related gene signatures portended worse survival in GBM patients (Extended Data Fig. 1e). These findings suggest that GSCs may co-opt neural developmental programs to support intercellular communication and promote glioma progression.

To dissect the role and mechanisms of TM formation in GSCs, we cultured GSCs in engineered cell culture units that permitted the selective isolation of TMs from GSCs, then performed proteomic and transcriptomic analyses. Protein synthesis-related processes, such as translation initiation, ribosomal subunit biogenesis, and RNA binding, were enriched in GSC TMs compared to the cell body (Extended Data Fig. 1f-h; Supplementary Table 1), indicating that GSC TMs possess molecular machinery to support localized protein synthesis. Neurons locally synthesize synaptic proteins at pre- and post-synaptic regions and within growth cones to permit receptor regulation, synaptic plasticity, and neurite-like structure maintance^36–39^. Local protein synthesis is important for axon guidance, elongation, survival, and regeneration^40–42^. Collectively, local translation serves essential roles in memory-based learning, plasticity, and cell survival in neurons, and its disruption has been linked to the onset of neurological diseases^43,44–46^. The striking similarities in the function of local protein synthesis in the normal nervous system with structural and functional aspects of TMs and associated networks led us to hypothesize that local protein synthesis in tumour microtubes supports multicellular communication and promotes GSC growth by enhancing intercellular crosstalk.

To evaluate local translation in GSC TMs and exclude the possibility that proteins were transported from the cell body, we first labelled endogenous RNAs by using the fluorescently labelled uridine-5′-triphosphate (Cy3-UTP). Cy3-UTP is incorporated into RNAs during synthesis, including rRNAs and mRNAs^47^, empowering visualization of fluorescent Cy3-RNA granules in GSC TMs. RNA tracing revealed that RNA granules moved into GSC TMs (Fig. 1d and Supplementary Video 1), facilitating assembly of local translation machinery within the TMs. Given the role of AMPARs in glioma cellular networks, we investigated the potential generation of AMPARs in TMs. We designed a translation reporter of AMPA glutamate receptor subunit by using the 5’ and 3’ untranslated regions (UTRs) of GluR1 fused to the photoconvertible fluorescent protein, Dendra2^48^. GSCs were transduced with this reporter and cultured in patterned structures to permit spatial orientation of cell bodies and TMs. We observed rapid, localized translation specifically at the TM tips upon laser stimulation (Fig. 1e, f), indicating that active translation occurred in GSC TMs. To validate this observation, we stained GSCs with O-propargyl-puromycin (OPP), an alkyne analog of puromycin that is efficiently incorporated into newly translated proteins to permit nascent protein production. Consistent with active local translation, we detected discrete OPP-positive puncta along GSC TMs (Extended Data Fig. 1i). These immunofluorescent results were validated with western blotting, in which GSC TMs showed greater OPP incorporation and enhanced capacity of local protein synthesis compared to DGCs, neurons, and astrocytes (Fig. 1g, h; Extended Data Fig. 1i), indicating that tumour cells require elevated protein production in GSC TMs to sustain network communication and survival compared with other cell types.

Given enrichment of neurotransmitter receptors and postsynaptic scaffolding proteins in GSC TMs (Extended Data Fig. 1b,1j), we investigated the role local protein synthesis in generating these proteins spatially by targeting translation in TMs. To restrict translation pharmacologic inhibition exclusively to TMs, we leveraged compartmentalized microfluidic devices with hydrostatic pressure barriers to prevent the diffusion of a translational inhibitor, cycloheximide (CHX), into the chamber containing the cell body (soma). Under these localized conditions, CHX treatment reduced the abundance of synaptic components within TMs (Extended Data Fig. 1j-l), suggesting that local translation is required for the assembly and maintenance of synapse-like structures. To evaluate the impact of translation inhibition on function of signalling in TMs, we assessed network connectivity after locally inhibiting protein synthesis and subsequently co-culturing the GSCs with partner tumour cells. While TM-mediated connections in vehicle controls facilitated the rapid retrograde propagation of calcium waves from partner cells (donors) to the GSCs (acceptors), CHX-treated TMs exhibited abrogated signal transmission (Fig. 1i and Supplementary Video 2). In parallel, we evaluated the response to neuronal cues and found that CHX treatment also markedly attenuated glutamate-evoked calcium influx (Fig. 1j). Collectively, these results demonstrate that TM-mediated connections are functionally dependent on local protein synthesis to sustain glioma network communication.

### Mitochondrial protein FASTKD2 maintains local protein synthesis and TM formation

To identify molecular targets involved in local protein synthesis in TMs, we interrogated our TM proteomic data, identifying mitochondrial-related pathways enriched in GSC TMs (Extended Data Fig. 1g). TMs contained more axon-like mitochondria than cell bodies, which correlated with more protein puncta (Extended Data Fig. 2a), in line with prior reports in which TMs display an abundance of mitochondria, including an axon-like rapid mitochondria transport system^7^. To test the role of mitochondria in TM local protein synthesis, GSCs were cultured to isolate TMs from their cell bodies, then TMs were treated with a series of metabolic inhibitors, and local protein synthesis measured. Mitochondrial inhibitors, including carbonyl cyanide-4 (trifluoromethoxy) phenylhydrazone (FCCP), which uncouples mitochondrial electron transport and disrupts the production of adenosine triphosphate (ATP), reduced TM protein synthesis more than glycolysis inhibitors (Extended Data Fig. 2b). Disrupting mitochondria specifically within TMs reduced ATP production throughout the cell (Extended Data Fig. 2c-f) and reduced tubule extension (Extended Data Fig. 2g).

**Figure 2.**
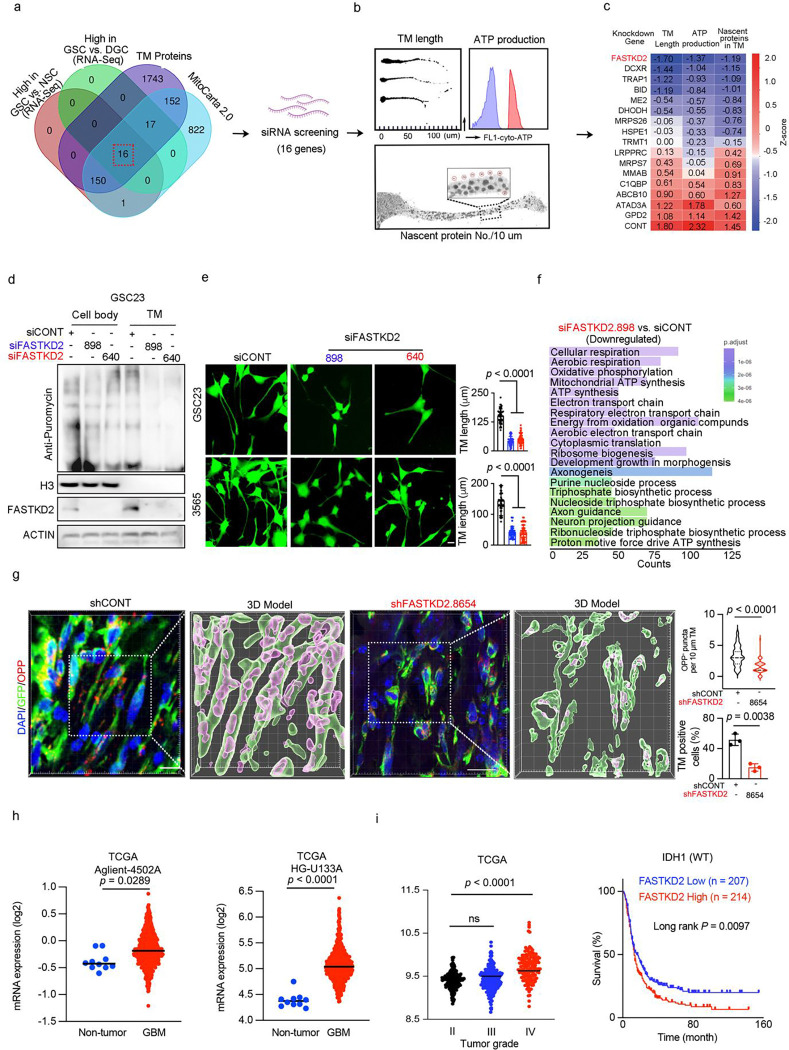
Mitochondrial protein FASTKD2 maintains local protein synthesis and TM formation a, A Venn diagram overlap illustrating the intersection between four datasets: 1) highly expressed differentially expressed genes (DEGs, padj < 0.05 and |log 2 (FoldChange)| > 1) in GSCs vs. NSCs (GSE41031); 2) highly expressed DEGs in GSCs vs. matched DGCs (GSE54791, padj < 0.05 and |log 2 (FoldChange)| > 1); 3) TM proteome from the studies above; and 4) mitochondria related genes as derived from MitoCarta 2.0. b, Schematic diagram of screen to identify candidate genes in GSCs that inhibit local protein synthesis initiation in TMs, ATP production, and TM length. c, Heatmap demonstrating Z-scores of nascent proteins, TM length, and the fluorescence intensity of ATP after genetic screening, respectively. d, GSCs were transduced with either a control siRNA (siCONT) or siFASTKD2, then cell body and TMs were collected and resolved by SDS-PAGE. TM lysates were probed with anti-puromycin antibody to determine the amount of local protein synthesis. ACTIN was used as a loading control. e, GSCs were transduced with eGFP and either a control siRNA (siCONT) or siFASTKD2, then TMs were imaged with immunofluorescence and length quantified. Scale bar: 40 μm. Right: Quantification of the TM length with and without siFASTKD2 knockdown. n = 50 TMs. f, GSCs were transduced with either a control siRNA (siCONT) or siFASTKD2, then TMs were collected, and RNA sequencing was performed. GO Enrichment Analysis of DEGs (padj < 0.05 and |log 2 (FoldChange)| > 1) between siFASTKD2 and siCONT in GSC23. g, GFP-labelled tumour cells with or without FASTKD2 knockdown were implanted into immunodeficient mice. Protein synthesis activity in tumour tissues was evaluated by in vivo OPP incorporation assay. Scale bar: 20 μm. h, FASTKD2 expression in non-tumour and GBM samples from the TCGA database is shown. i, FASTKD2 expression in human non-tumour brain tissues and different subtypes of GBM tumour samples from the TCGA database is shown. j, Kaplan–Meier overall survival of IDH-wildtype patients from the CGGA database stratified by median FASTKD2 expression. High expression n = 207 and low expression n = 214. **, p = 0.0097 by log-rank test. k, The CGGA dataset was examined for correlation between expression of FASTKD2 and a gene signature for regulation of communication by electrical coupling in IDH wildtype glioblastoma. l, The CGGA dataset was examined for correlation between expression of FASTKD2 and a gene signature for positive regulation of synapse assembly in IDH wildtype glioblastoma. m, Combined Kaplan–Meier survival analysis of FASTKD2 expression and the regulation of communication by electrical coupling pathway in the CGGA. n, Combined Kaplan–Meier survival analysis of FASTKD2 expression and GAP junction in the CGGA. Representative of two independent experiments in j. Data are presented from three independent experiments in g, I, and k. Data are presented as mean ± SD. two-tailed Student’s t-test with p values for g. Two-way ANOVA followed by multiple comparisons with adjusted p-values for i. One-way ANOVA followed by multiple comparisons with adjusted p-values for k.

Next, we conducted intersection analysis of the GSC TM proteome and mitochondria-related proteins derived from the MitoCarta 2.0 database. Selecting mitochondrial proteins highly expressed in GSCs relative to both DGCs and NSCs, we identified 16 overlapping mitochondrial proteins enriched in GSCs (Fig. 2a and Supplementary Table 2). We designed a functional screen of these 16 targets in TM biology using a custom siRNA library. The use of siRNAs, instead of shRNAs or sgRNAs, permitted direct targeting of gene expression without the need for the transcriptional machinery of the cell body. We used three complementary readouts of modulating TMs: 1) local protein synthesis, 2) ATP production in TMs, and 3) tubule length in GSCs (Fig. 2b; Supplementary Table 3) and identified Fas Activated Serine-Threonine Kinase Domains 2 (FASTKD2) (Fig. 2c). FASTKD2 protein puncta were higher in abundance along GSC TMs than in the cell body (Extended Data Fig. 2h). FASTKD2 knockdown suppressed local protein synthesis in TMs compared to cell body (Fig. 2d) and reduced their formation (Fig. 2e), suggesting that FASTKD2 is spatially enriched at sites of active translation and may be required to maintain local protein synthesis within TMs. Indeed, RNA sequencing analysis after FASTKD2 knockdown in GSCs revealed downregulation of pathways associated with translation, ATP synthesis, and axonogenesis (Fig. 2f). Consistent with the in vitro findings, in vivo analysis revealed that FASTKD2 knockdown reduced both protein synthesis within TMs and TM formation (Fig. 2g). These findings support FASTKD2 as a spatially localized regulator that integrates metabolic and translational control to maintain TM function.

To determine the potential clinical relevance of FASTKD2, we examined publicly available datasets; FASTKD2 was preferentially expressed in human GBM surgical specimens relative to non-tumour brain tissues (Fig. 2h). FASTKD2 gene expression portended poor patient survival in the Chinese Glioma Genome Atlas (CGGA) database (Fig. 2i). FASTKD2 expression positively correlated with AMPARs and pathways associated with synaptic assembly and neuron–glioma communication (Extended Data Fig. 3a, b), and we also observed a positive association of FASTKD2 with other neuronal receptors, like acetylcholine receptors (data not shown). Combined survival analysis incorporating FASTKD2 and communication-related gene signatures reinforced prognostic significance of FASTKD2 in the context of neural activation (Extended Data Fig. 3c). Together, these findings identify FASTKD2 as a potential therapeutic target for disrupting intercellular communication.

### FASTKD2 maintains cancer stemness

**Figure 3.**
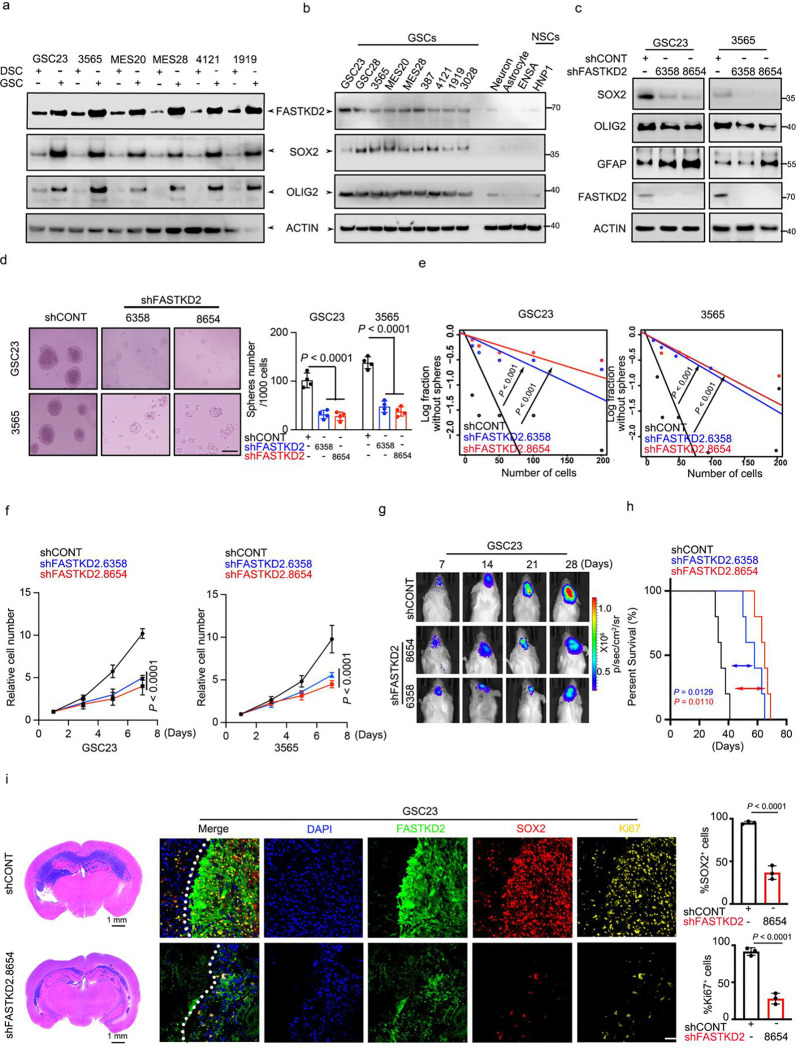
FASTKD2 maintains GSCs and promotes tumour growth. a, Protein expression levels of FASTKD2 and GSC markers (SOX2 and OLIG2) were assessed by western blot in matched pairs of patient-derived GSCs and DGCs. ACTIN was used as a loading control. b, Protein expression levels of FASTKD2 and GSC markers (SOX2 and OLIG2) were assessed by western blot in GSCs, normal neural stem cells (ENSA, HNP1, and NSC11), and human astrocytes, ACTIN was used as a loading control. c, GSCs were transduced with either a control shRNA (shCONT) or one of two shFASTKD2s, then lysates were derived and resolved by SDS-PAGE. Protein levels of stemness markers (SOX2 and OLIG2) were assessed by immunoblot. ACTIN served as a loading control. d, GSCs were transduced with either a control shRNA (shCONT) or one of two shFASTKD2s, then cultured in nonadherent serum-free conditions. Left: Representative tumour sphere formation. Scale bar: 50 μm. Right: Quantification of tumour spheres. N = 4. e, GSCs were transduced with either a control shRNA (shCONT) or one of two shFASTKD2s, then cultured with under in vitro extreme limiting dilution. N = 3 independent biological cell cultures. f, GSCs were transduced with either a control shRNA (shCONT) or one of two shFASTKD2s, then cell viability was measured. Data represent mean ± SD. N = 3. g, GSCs were transduced with a bioluminescence reporter and either a control shRNA (shCONT) or one of two shFASTKD2s, then implanted into the brains of immunocompromised mice. Bioluminescent in vivo IVIS imaging was performed weekly. N = 5 mice per group. h, Kaplan–Meier survival curves of mice implanted with GSC23 transduced with either shCONT or shFASTKD2. N = 5 mice per group. i, Immunofluorescent staining and quantification of SOX2 (red) and Ki67 (cyan) in GSC-derived xenografts from mice bearing GSCs transduced with shCONT and shFASTKD2. N = 3 random fields from triplicate samples. Scale bar: 40 μm. Representative of two independent experiments in a, b and c. Data are presented from three independent experiments in d, e, f, and i. Data are presented as mean ± SD. Student’s t-test with p values for i. Two-way ANOVA followed by multiple comparisons with adjusted p-values for f. One-way ANOVA followed by multiple comparisons with adjusted p-values for d. Log-rank test for h. Extreme limiting dilution assay (ELDA) analysis for e.

To investigate FASTKD2 contributions to GSC maintenance, we first examined its expression across GSCs, DGCs, and NSCs. FASTKD2 was expressed at higher levels in GSCs than matched DGCs or NSCs (Extended Data Fig. 3d, e). Active enhancer marker histone 3 lysine 27 acetyl (H3K27ac) showed greater enrichment at the FASTKD2 promoter in GSCs compared to NSCs (Extended Data Fig. 3f). Patient-derived GSCs cultured in vitro expressed higher FASTKD2 protein levels compared to matched DGCs or NSCs (Fig. 3a, b). FASTKD2 mRNA levels positively correlated with expression levels of GSC markers, including SOX2 and CD44, in publicly available datasets (Extended Data Fig. 3g). To assess FASTKD2 function in GSCs, patient-derived GSCs were transduced with either one of two shRNAs encoding non-overlapping sequences targeting FASTKD2 (shFASTKD2.6358 or shFASTKD2.8654, in which the number designates the start site) or a control shRNA that does not target a known mammalian gene (shCONT), revealing that FASTKD2 knockdown decreased in vitro expression of stem cell markers, SOX2 and OLIG2 (Fig. 3c) and self-renewal measured by number of tumour spheres formed (Fig. 3d) and in vitro limiting dilution (Fig. 3e). Silencing FASTKD2 reduced in vitro GSC proliferation compared to NSCs and DGCs (Fig. 3f and Extended Data Fig. 3h-j). GSCs expressing a bioluminescence reporter were transduced with either shFASTKD2 or shCONT and then implanted into the brains of immunocompromised mice and monitored for tumour growth. FASTKD2 knockdown reduced tumour flux as measured by bioluminescent imaging (Fig. 3g), which translated into increased survival of mice bearing GSCs transduced with shFASTKD2 relative to those with shCONT (Fig. 3h). Brains harvested from mice xenografted with shFASTKD2-expressing GSCs showed reduced tumour burden, as measured by histologic staining, and decreased GSC frequency, as measured by SOX2 immunofluorescence (Fig. 3i). Collectively, FASTKD2 maintains tumour stemness in vitro and in vivo.

### FASTKD2 facilitates glioma network communication through local protein synthesis in TMs

**Figure 4.**
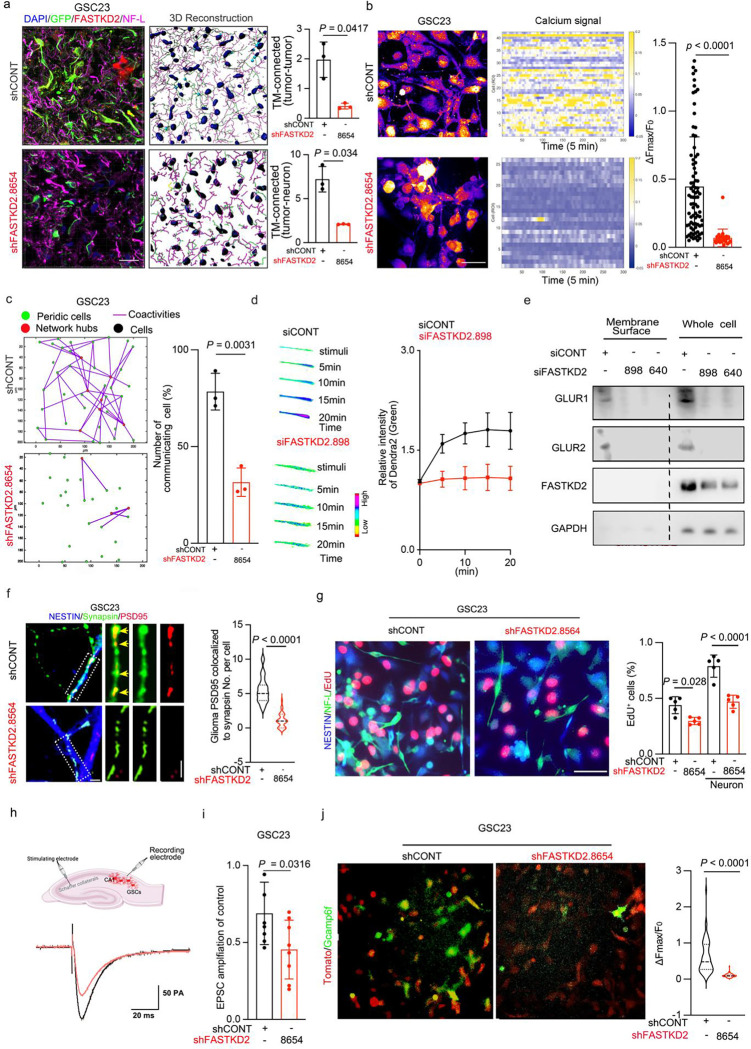
FASTKD2 facilitates neuronal-tumour cell connectivity through local protein synthesis in TMs a, Representative immunofluorescence images of mouse tumour sections under linezolid treatment and tumour cell labelled with GFP (Green), neuron marker [NF-L (Red)], and general marker of nuclei [DAPI (Blue)]. Representative images are shown. Scale bar: 40 μm. Middle: 3D reconstruction of tumour cell–tumour cell and tumour cell–neuron networks. Right: quantitative analysis of tumour cell–tumour cell and tumour cell–neuron interactions. N = 3 fields per slice from n = 3 slices. b, Left: representative fluorescence images of intracellular Ca^2+^ in GSC23 cells. Middle: heatmap of single-cell Ca^2+^ signals over time. Right: quantification of the maximal Ca^2+^ response (ΔFmax/F_0_) in GSC23 cells with or without FASTKD2 knockdown. c, Representative functional Ca^2+^ in vitro coactivity networks in GSC23 cells transduced with shCONT (top) or FASTKD2 knockdown (shFASTKD2.8654, bottom) . Each dot represents an individual cell; green nodes indicate periodic cells, and red nodes mark network hubs (top 5% highest degree). Purple lines represent significant coactivities (r ≥ 0.15), and edge lengths are scaled to spatial cell–cell distances. Scale bar, 40 μm. Right: quantification of the percentage of communicating cells (i.e., cells involved in at least one significant coactivity). Data represent mean ± s.e.m. from three independent fields. d, GSC23 was transfected with 5’UTR_GluR1_-Dendra2–3’UTR _GluR1_ to evaluate local translation upon transfection with either siCONT or siFASTKD2. Left: Pseudo-colour images (green) of locally synthesized Dendra2 reporter in terminals of tubules after siCONT or siFASTKD2. Right: Quantification of newly synthesized Dendra2 reporters in the axonal tip after siCONT or siFASTKD2 transduction. N = 3 replicates. e, GSC23 was transfected with either siCONT or one of two siFASTKD2s, then lysates were generated and resolved by SDS-PAGE. Protein levels of AMPA receptors (GluR1 and GluR2) and FASTKD2 in cell membranes and whole cell were assessed by immunoblot. ACTIN was used as a loading control. f, GSC23 was transfected with either shCONT or one of two shFASTKD2s, then co-cultured with neurons. Cultures were assessed for stemness (NESTIN), presynaptic puncta (synapsin, green) and postsynaptic puncta (PSD95, red) by immunofluorescence. Left: Representative images are shown. Scale bar: 5 μm. Right: Quantification of post-synaptic glioma-derived PSD95 colocalized with neuronal pre-synaptic synapsin in co-cultures. N = 30. g, Representative immunofluorescence images (left) and proliferation index quantification (right; EdU^+^ cells/Nestin^+^ cells) of glioma cells (GSC23) cultured alone or with neurons in with or without FASTKD2 knockdown. Scale bar: 50 μm. h, Left: Electrophysiological model created by Biorender; GFP^+^ glioma xenografted in hippocampal CA1; Representative traces of EPSC in xenografted glioma with shCONT and shFASTKD2 glioma cells. i, Quantification of current amplitude from (i). N = 7 cells, 5 mice (shCONT) and 7 cells, 5 mice (shFASTKD2). j, Left: Calcium imaging (120 second time course) of hippocampal slice from mice xenografted with GCaMP6f-expressing GSCs. Green denotes glioma GCaMP6f and red denotes dTomato. n = 3 mice per group. Scale bar: 50 μm. Right: Calcium peaks per minute per 100 cells was quantified. N = 5 fields. Representative of two independent experiments in d. Data are presented from three independent experiments in a, c, f, i, and j. Data are presented as mean ± SD. Two-tailed Student’s t-test with p-values for a-c, f, i, and j. One-way ANOVA followed by multiple comparisons with adjusted p-values for g.

TM networks facilitate long-range communication of glioma cell by intercellular Ca^2+^ waves^7,8^, which are used for directed tumour self-repair and better cellular homeostasis. TM networks also receive synaptic neuronal input that activates glioma network communication, further driving glioma invasion and proliferation^49,50^. Based on this background, knockdown of FASTKD2 markedly disrupted both tumour cell–tumour cell and neuron–tumour cell connections (Fig. 4a). FASTKD2 knockdown reduced spontaneous Ca^2+^ transients compared to controls. To better understand the patterns of intercellular Ca^2+^ communication in glioma, we employed mathematical network analysis, defining nodes as individual cells and functional edges as spatiotemporally synchronized calcium events (detected within 100 μm, timeframe = 1 s). Network reconstruction unveiled a hierarchical architecture driven by a discrete subpopulation of ‘periodic cells’ that display autonomous rhythmicity in control group. These cells function as high-connectivity hubs to orchestrate global intercellular co-activity. FASTKD2 depletion eliminated rhythmic hubs and abrogating network synchronization (Fig. 4b, c and Supplementary Video 3). Together, these data establish FASTKD2 as the essential driver of the functional network hierarchy.

FASTKD2 depletion impaired the synthesis of neuronal receptors (Fig. 4d) and disrupted AMPAR membrane trafficking (Fig. 4e). To determine whether FASTKD2 contributes to glioma-neuron synapse formation and neuronal signal transmission, we examined its impact on glioma-neuron connectivity. Neurons co-cultured with GSCs transduced by shFASTKD2 formed fewer synaptic connection compared to co-culture with GSCs transduced by shCONT as indicated by decreased co-localization of neuronal presynaptic puncta (synapsin) with glioma postsynaptic puncta (PSD95) (Fig. 4f) and tumour cell proliferation (Fig. 4g). To validate these observations, we transduced GSCs with a calcium reporter, GCaMP6f (which has a fluorescent reporter of single neuronal action potentials with fast response kinetics)^51^, and either shCONT or shFASTKD2, then implanted the transduced gliomas cells into hippocampi and stimulated the axonal afferents (Schaffer collaterals) into the CA1 region of the hippocampus bearing transduced glioma cells. Axonal stimulation elicited inward currents in xenografted glioma cells, which were reduced by FASTKD2 knockdown (Fig. 4h, i). Intercellular calcium signalling was effectively silenced in FASTKD2-depleted networks (Fig. 4j and Supplementary Video 4), Collectively, these results demonstrate that FASTKD2 maintains structural and functional integrity required for glioma cells to integrate into and communicate within multicellular networks.

### FASTKD2 maintains mitochondrial function and ATP production in GSC TMs

The FASTKD family encodes for RNA-binding proteins that regulate mitochondrial function. FASTKD2 is a mitochondrial protein essential for oxidative phosphorylation (OXPHOS) through its regulation of post-transcriptional control of mitochondrial gene expression^52,53^. Therefore, we hypothesized that the loss of FASTKD2 induced mitochondrial dysfunction within GSC TMs, resulting in a failure of ATP production and local protein synthesis. Consistent with this hypothesis, FASTKD2 knockdown in GSCs downregulated mitochondrial gene expression, mitochondrial function, and mitochondrial respiratory chain pathways by GSEA (Extended Data Fig. 4a). Expression levels of key mitochondrial genes, including ATP8, CO1, CO2, ND1, ND2, and ND6, were markedly decreased upon FASTKD2 knockdown (Extended Data Fig. 4b), associated with inhibition of de novo protein synthesis within the mitochondria of GSC TMs (Extended Data Fig. 4c) and collapse of the mitochondrial respiratory chain (Extended Data Fig. 4d)，This was associated with disruption of the TCA cycle (Extended Data Fig. 4e) and a marked reduction in mitochondrial motility within GSC TMs (Extended Data Fig. 4f), ultimately resulting in decreased ATP production compared to TMs derived from GSCs transduced with siCONT (Extended Data Fig. 4g, h). Collectively, FASTKD2 promotes mitochondrial function and ATP homeostasis within TMs.

### FASTKD2 binds YTHDF2 to stabilize mitochondrial ribosomal RNA and maintain mitochondrial function

**Figure 5.**
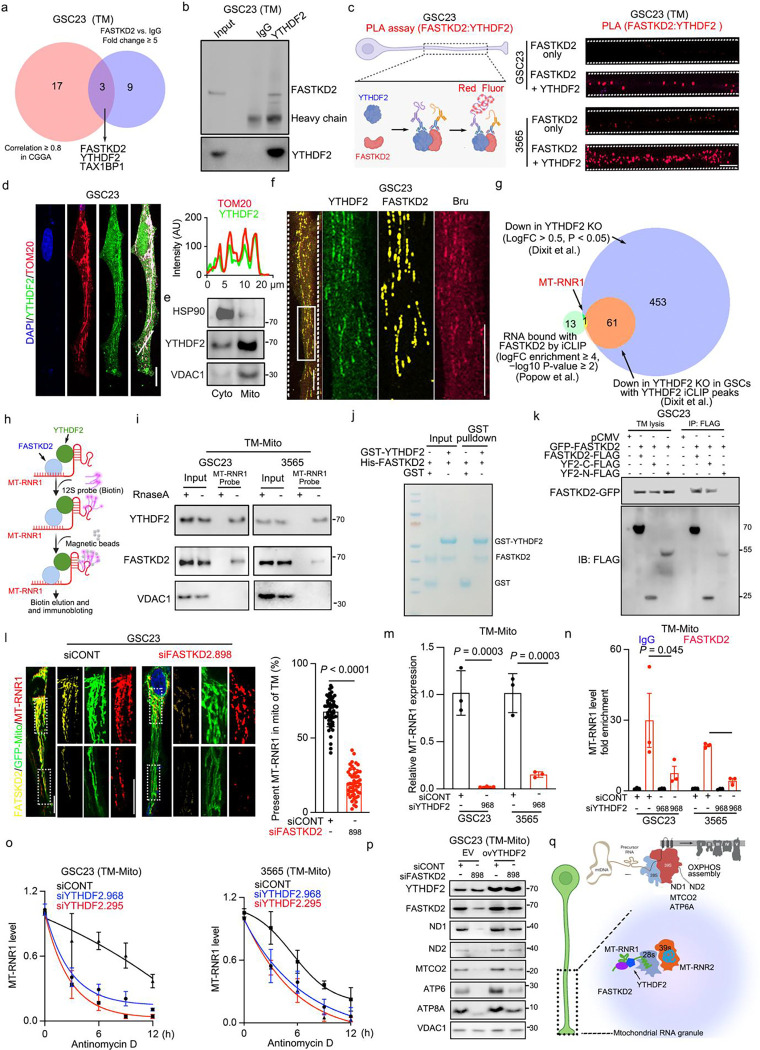
FASTKD2 maintains mitochondrial function through YTHDF2 stabilization of MT-RNR1 a, Venn diagram depicting the intersection of proteins detected by mass spectrometry ≥ fivefold levels upon immunoprecipitation by anti-FASTKD2 antibody relative to IgG control and genes whose expression correlated with FASTKD2 with coefficient ≥ 0.8 in the CGGA. b, Lysates from GSC TMs underwent co-IP with either anti-YTHDF2 or IgG control followed by immunoblotting for FASTKD2 and YTHDF2. c, Left: A schematic diagram of proximity ligation assay used to evaluate the interaction between FASTKD2 and YTHDF2. Right: Representative imaging of the interaction between YTHDF2 and FASTKD2 in TMs. Scale bar: 20 μm. d, Left: Immunofluorescent imaging depicting the co-localization between YTHDF2 (green) and mitochondria (TOM20, red) in TMs. Scale bar: 20 μm. Right: Co-localization intensity analysis of YTHDF2 and TOM20. e, TMs from GSCs were separated into cytoplasmic and mitochondrial fractions, and lysates resolved by SDS-PAGE. Immunoblotting for cytoplasm Western blot analysis indicating the presence and expression of YTHDF2 in mitochondria of GSC TMs. f, Immunofluorescent imaging of the co-localization between YTHDF2 (green), FASTKD2 (yellow), and newly synthesized RNA (bromouridine, BrdU (red)) in TMs. Scale bar: 20 μm. g, Venn diagram illustrating overlapping genes among three sets: genes downregulated upon YTHDF2 knockout GSCs (PMID: 33023892)^76^, RNA binding to FASTKD2 identified by iCLIP in vitro (PMID: 26370583)^53^, and genes downregulated after YTHDF2 knockout in GSCs with YTHDF2 iCLIP peaks (PMID: 33023892)^76^. h, Schematic of ChiRP (Chromatin Isolation by RNA purification) with MT-RNR1 followed by immunoblotting for FASTKD2 and YTHDF2. i, GSC TMs were purified, then input lysates and ChIRP for MT-RNR1 RNA immunoprecipitated by DNA probes against MT-RNR1 were treated with vehicle control or RNase followed by immunoblotting for FASTKD2, YTHDF2, and mitochondrial marker (VDAC1). j, GST-pull-down in GSCs confirmed the interaction between YTHDF2 and FASTKD2 in vitro. k, GSCs were transduced with vector control (pCMV) or full-length GFP-tagged FASTKD2 and FLAG-tagged full length or C- or N-terminal domains of YTHDF2. Left lanes show input lysates. Right lanes show FLAG-immunoprecipitation followed by immunoblotting for GFP and FLAG. l, GSCs were transduced with either control siRNA (siCONT) or siFASTKD2. Left: Representative images of MT-RNR1 detected by FISH assay and FASTKD2 (yellow) and mitochondria (green) by immunofluorescence. Scale bar: 20 μm. Right: Quantification of the percentage of MT-RNR1 in the mitochondria of GSC TMs. (siCONT, n = 52; siFASTKD2, n = 50). m, GSCs were transduced with either control siRNA (siCONT) or siYTHDF2. Relative RNA expression of MT-RNR1 and MT-RNR2 in whole cell lysates of GSCs was measured by PCR. N = 3. n, GSCs were transduced with either control siRNA (siCONT) or siYTHDF2. MT-RNR1 fold enrichment in GSC TMs as detected by RNA immunoprecipitation followed by quantitative PCR (RIP-qPCR). N = 3. o, Time course of changes in the mRNA levels of MT-RNR1 following actinomycin D treatment in GSCs transduced with siCONT or siYTHDF2. N = 3. p, GSCs (GSC23) was transduced with either empty vector (EV) or YTHDF2 in combination with siCONT or siFASTKD2, then lysates were generated from TM mitochondria and subjected to SDS-PAGE. Immunoblotting for YTHDF2, FASTKD2, and mitochondrial proteins was performed. VDAC1 served as a loading control. q, Proposed mechanistic diagram of YTHDF2 and FASTKD2 regulating mitochondrial gene expression in GSC TMs. Representative of two independent experiments in b, e, I, and k. Data are presented from three independent experiments in m-o. Data are presented as mean ± SD. two-tailed Student’s t-test with p-values for l. Two-way ANOVA followed by multiple comparisons with adjusted p-values for o. One-way ANOVA followed by multiple comparisons with adjusted p-values for m-n.

To investigate downstream molecular mechanisms by which FASTKD2 maintains mitochondrial function in TMs, we purified TMs derived from GSCs, then immunoprecipitated binding partners of FASTKD2 through mass spectrometry. Within the FASTKD2 interactome, the m^6^A reader YTHDF2 was the top interactor (Fig. 5a; Supplementary Table 4). FASTKD2 and YTHDF2 binding was confirmed by both immunoprecipitation and proximity ligand assays (Fig. 5b, c). Both FASTKD2 and YTHDF2 bind RNA and are involved in mitochondrial RNA processing, mitochondrial-encoded protein synthesis, local translation, and localization of neuronal mRNAs. The convergence of biochemical function led us to hypothesize that YTHDF2 and FASTKD2 collaboratively promote mitochondrial RNA processing or transport to enhance mitochondrial function in GSC TMs. Therefore, we explored YTHDF2 localization in GSC TMs. YTHDF2 localized to mitochondria and co-localized with mitochondrial RNA granules in GSC TMs assessed by immunofluorescence and western blotting (Fig. 5d-f). Among YTHDF family members, YTHDF2 was uniquely localized to TM mitochondria in GSCs, in contrast to YTHDF1 and YTHDF3 (Extended Data Fig. 5a). Concordantly, YTHDF2 knockout downregulated expression of mitochondrial respiratory chain complexes and pathways related to mitochondrial ribosomes by RNA sequencing (Extended Data Fig. 5b-d).

Given that FASTKD2 and YTHDF2 both bind RNA, we sought RNAs that bound both proteins as a possible mechanism of interaction. Therefore, we interrogated available assays, including separate results measuring RNA binding by FASTKD2 and YTHDF2 from individual-nucleotide resolution UV crosslinking and immunoprecipitation (iCLIP), then considered shared RNAs. FASTKD2 and YTHDF2 intersection analysis identified the mitochondrial RNA ribosomal subunit, MT-RNR1, as the only common interacting RNA molecule. MT-RNR1 is a mitochondrially encoded 12S ribosomal RNA and a component of the small subunit of the mitochondrial ribosome (Fig. 5g). FASTKD2 and YTHDF2 binding to MT-RNR1 was greater in mitochondria located in GSC TMs compared to cell bodies by RNA immunoprecipitation (RIP)-PCR (Extended Data Fig. 5e-g). Based on these results, we hypothesized that YTHDF2 and FASTKD2 co-bound MT-RNR1 in mitochondrial RNA granules (MRGs) to maintain mitochondrial gene expression in TMs. To test this, we performed Chromatin Isolation by RNA Purification (ChIRP) using the 12s (MT-RNR1) probe (Fig. 5h) in GSC TMs, revealing binding to YTHDF2 and FASTKD2 (Fig. 5i). The interaction between FASTKD2 and YTHDF2 persisted after RNase treatment (Extended Data Fig. 5h), suggesting that MT-RNR1 was not essential to the binding between YTHDF2 and FASTKD2 but rather was direct. To validate the interaction between FASTKD2 and YTHDF2 in vitro, His-tagged recombinant FASTKD2 was incubated with or without recombinant GST-tagged YTHDF2 under cell-free conditions, then pulldown assays confirmed direct binding of YTHDF2 to FASTKD2 (Fig. 5j). YTHDF2 has a bipartite domain architecture, characterized by a P/Q/N-rich N-terminal domain (YTHDF2-N) and a C-terminal YTH domain (YTHDF2-C). To determine YTHDF2 domains that interact with FASTKD2 in TMs, GSCs were transfected with GFP-tagged FASTKD2 and FLAG-tagged YTHDF2 constructs (full length YTHDF2, YTHDF2-C-FLAG, or YTDHF2-N-FLAG), then TMs purified and immunoprecipitated to test for interactions using the GFP and FLAG tags. FASTKD2 interacted with full-length and C-terminal YTHDF2 constructs but showed limited biding to N-terminal YTHDF2 (Fig. 5k).

As FASTKD2 binds RNAs localized in MRGs and slides along mitochondrial networks^54^ to regulate mitochondrial gene expression^53,54^, we examined its role in MT-RNR1 localization. FASTKD2 knockdown did not alter MT-RNR1 expression at the whole cell level (Extended Data Fig. 5i) but reduced MT-RNR1 in TMs, particularly in the distal TM compared to the proximal TM (Fig. 5l and Extended Data Fig. 5j), suggesting that FASTKD2 regulates MT-RNR1 transport along TMs to support mitochondrial gene expression. To visualize this process, we generated GSCs with stable expression of eGFP-tagged FASTKD2, then monitored GFP movement within TMs. Live imaging revealed that FASTKD2 moved dynamically along the mitochondrial network in TMs (Extended Data Fig. 5k and Supplementary Video 5). Mitochondria within TMs were highly elongated (Fig. 5l and Extended Data Fig. 2a). The number of MRGs has been reported to correlate with mitochondrial length^54^, so GSCs may store more granules and facilitate MRG movement along mitochondrial networks to promote mitochondrial-encoded protein synthesis and ultimately mitochondrial function in TMs.

Phenocopying FASTKD2 knockdown, YTHDF2 knockdown reduced MT-RNR1 expression in GSCs (Fig. 5m) and binding between FASTKD2 and MT-RNR1 (Fig. 5n). YTHDF2 knockdown also decreased MT-RNR1 stability (Fig. 5o) and impaired mitochondrial protein synthesis in TMs (Extended Data Fig. 5l). In rescue studies, YTHDF2 overexpression partially rescued the defects in mitochondrial protein expression and synthesis in GSC TMs caused by FASTKD2 knockdown (Fig. 5p and Extended Data Fig. 5m). Collectively, FASTKD2 enables efficient transport of MT-RNR1 along mitochondrial networks in the TM, while YTHDF2 promotes MT-RNR1 stability. Together, the FASTKD2-YTHDF2-MT-RNR1 axis drives mitochondrial gene expression and promotes local protein synthesis in the TM of GSCs.

### Identification of linezolid as a disruptor of FASTKD2 binding to MT-RNR1

**Figure 6.**
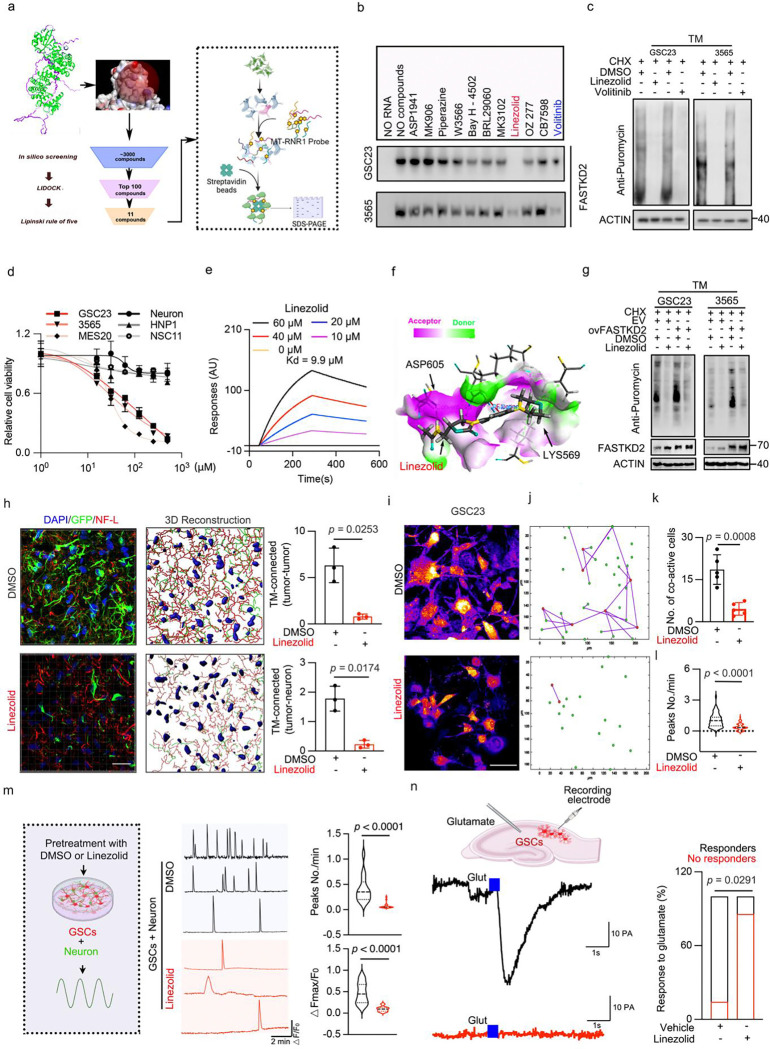
Pharmacological targeting FASTKD2 suppresses neuronal-glioma interactions and GSC proliferation. a, Flowchart depicting experimental design for FASTKD2 drug screening in GSCs based on blockade of binding to MT-RNR1. Left: In silico screening flowchart. Right: Flowchart depicting in vitro RNA immunoprecipitation followed by western blotting (RIP-WB) experimental design to screen compounds. b, Two patient-derived GSCs were treated with DMSO or a panel of top hits from (a) then subjected to RIP-WB to examine the affinity between compound hits from panel A and intracellular FASTKD2. c, GSCs were treated with vehicle control (DMSO), linezolid, or volitinib for 48 hours, then cycloheximide (CHX, 20 μg/ml) was added for 20 minutes prior to puromycin treatment for 15 minutes. Lysates were derived, and immunoblotting was performed to analyse mitochondrial protein synthesis under indicated drug treatment in GSCs. Purified mitochondrial lysates were probed with anti-puromycin antibody to measure the ability of mitochondrial protein synthesis. d, Cell viability assay to examine the inhibitory effects on GSCs (GSC23, 3565, and MES20), neural stem cells (NSC11 and HNP1), and neurons after treatment with increasing concentrations of linezolid for 48 hours. e, Surface plasmon resonance was used to analyse binding affinities and kinetic parameters between FASTKD2 and increasing concentrations of linezolid. f, Molecular docking using Discovery Studio software was used to analyse the interaction between FASTKD2 and linezolid. g, GSCs were transduced with an empty vector (EV) or FASTKD2 overexpression then treated with vehicle control (DMSO) or linezolid for 48 hours. TM lysates were probed with anti-puromycin antibody to measure the ability of local protein synthesis as measured by western blotting. h, Representative immunofluorescence images of mouse tumour sections under linezolid treatment and GSC23 tumour cell label with GFP (Green), neuron marker [NF-L (Red)], and general marker of nuclei [DAPI (Blue)]. Representative images are shown. Scale bar: 40 μm. Middle : 3D reconstruction of tumour–tumour and tumour–neuron networks. Right: quantitative analysis of tumour–tumour and tumour–neuron interactions in the presence or absence of linezolid treatment. N=3 tumours, n=3 h.p.f. i, Representative fluorescence images showing intracellular Ca^2+^ in GSC23 cells in the presence or absence of FASTKD2 knockdown. j, Network architecture of co-active cell pairs derived from Ca2+ recordings of GSC23 tumour cells with or without FASTKD2 knockdown in vitro. k, Quantification of the percentage of communicating cells (i.e., cells involved in at least one significant coactivity) in GSC23 with or without FASTKD2 knockdown. n = 4 random fields from triplicate samples. l, Quantification of calcium peaks n number in GSC23 in the presence of linezolid treatment. m, Schematic with GSC23 co-cultured with neurons with treatment by DMSO or linezolid, then currents in glioma cells were monitored. Centre: Representative tracings are shown. Right: Quantification of peaks number and the maximal Ca^2+^ response (ΔFmax/F_0_) in GSC23 cells. n, Representative traces of glutamate (blue)-evoked currents in GSC23 xenografted glioma. Top, glutamate responder cell. Bottom, non-responder cell. Right, Quantification of glutamate responders and non-responders. N = 7 vehicle group glioma cells from 5 mice and 7 linezolid treated with glioma cells from 5 mice. Representative of two independent experiments in b, c and g. Data are presented from three independent experiments in d, h, I, and k-n. Data are presented as mean ± SD. Two-tailed Student’s t-test with p-values for h and k-n.

To translate our findings into potential preclinical therapeutic paradigms, we sought pharmacologic agents to target FASTKD2 functional through disruption of its binding to MT-RNR1 by sequential in silico and in vitro drug screens of known compounds to accelerate potential clinical trial development. The RAP domain of FASTK family members shares structural similarities with PD-(D/E) XK nucleases (Extended Data Fig. 6a)^55,56^. We aligned nuclease structures with the FASTKD2 structure predicted by AlphaFold2.0 and found that the RAP domain of FASTKD2 closely resembled a PD-(D/E) XK nuclease (Extended Data Fig. 6b). We then utilized this model to perform in silico drug screening with an FDA-approved drug library and identified 11 potential candidate compounds (Fig. 6a; Supplementary Table 5). We prioritized targeting FASTKD2 binding to MT-RNR1 as this appears to be most directly associated with the mitochondrial function, so we performed RNA immunoprecipitation with western blotting (RIP-WB) to evaluate the impact of these 11 candidate drugs on binding interactions between MT-RNR1 and intracellular FASTKD2. Linezolid and volitinib showed the greatest effect on inhibiting binding affinity (Fig. 6b) and mitochondrial protein synthesis (Fig. 6c). Linezolid inhibited GSC proliferation at lower drug concentrations than NSCs and neurons grown in vitro, in contrast to volitinib (Fig. 6d and Extended Data Fig. 6c). Therefore, we prioritized linezolid as our lead drug compound in additional experiments. To confirm the interaction between FASTKD2 and linezolid, surface plasmon resonance assays measured the binding affinity of linezolid to FASTKD2 with a Kd of 9.9 μM (Fig. 6e). Cellular thermal shift assays demonstrated that linezolid stabilized intercellular FASTKD2 (Extended Data Fig. 6d, e). To ascertain the mode of the binding, we visualized the binding site of linezolid on FASTKD2 using computational docking and predicted key interactions of linezolid through hydrogen bonding with FASTKD2 at amino acids ASP605 and LYS569 (Fig. 6f). Based on these predictions, we engineered a mutation into the ASP605 site of FASTKD2 and found that linezolid bound to the mutant FASTKD2 with lower affinity than wildtype FASTKD2 (Extended Data Fig. 6f-i), suggesting that this site contributed to drug binding. To confirm the functional contribution of FASTKD2 in effects of linezolid, we performed rescue studies by overexpressing FASTKD2 with linezolid treatment and measured mitochondrial protein synthesis by OPP in GSCs, which was partially rescued by FASTKD2 overexpression (Fig. 6g). Thus, linezolid is an FDA-approved agent that may be repurposed to target FASTKD2 tumour functions.

### Linezolid suppresses glioma network communication in vitro and in vivo

To determine the on-target, mechanistic effects of linezolid, we investigated the effects on cellular phenotypes elicited by genetic targeting of FASTKD2. Linezolid treatment of GSCs inhibited ATP production (Extended Data Fig. 6j), TM formation (Extended Data Fig. 6k) and GluR1 expression (Extended Data Fig. 6l). Like FASTKD2 knockdown, linezolid treatment disrupted tumour cell–tumour cell and neuron–tumour cell connectivity mediated by TMs (Fig. 6h), leading to a pronounced attenuation of Ca^2+^ communication within the TM-connected network (Fig. 6i-l). To further evaluate TM disruption in neuron–glioma communication, we co-cultured neurons and GSCs. Linezolid reduced the formation of postsynaptic densities at neuron–tumour interfaces (Extended Data Fig. 7a) and decreased the frequency of neuronally evoked neurotransmission glioma cells (Fig. 6m), resulting in loss of neuron-induced glioma proliferation (Extended Data Fig. 7b).

Linezolid has been used as an antibiotic for the treatment of severe infections, including those of the central nervous system, because it has intracranial penetration, suggesting potential utility in the treatment of central nervous system malignancies under drug repurposing. Therefore, we investigated effects of linezolid on neuronal-tumour crosstalk in vivo. GSCs were xenografted into hippocampi and tumour responses to glutamate were reduced with linezolid treatment (Fig. 6n). GSCs transduced with the GCaMP6f calcium reporter were engrafted into the brains of immunocompromised mice and allowed to integrate. Following treatment with linezolid or vehicle, we performed ex vivo calcium imaging on acute brain slices. Linezolid treatment suppressed global calcium activity, indicating disruption of TM-mediated communication networks (Extended Data Fig. 7c-e and Supplementary Video 6). Thus, linezolid treatment phenocopies the effects of FASTKD2 depletion, compromising glioma network integrity and severing neuron–glioma communication to suppress tumour proliferation.

### Linezolid inhibits in vivo tumour growth

**Figure 7.**
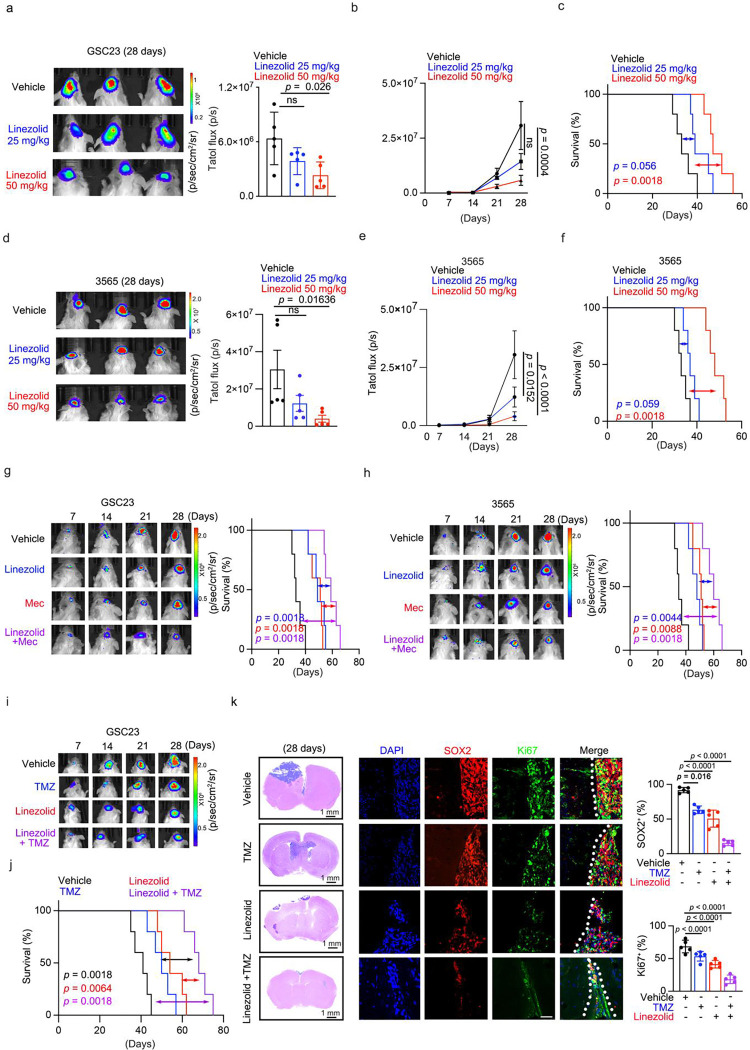
Combined inhibition of neuron–glioma connectivity augments efficacy against glioblastoma a, Left: Representative in vivo bioluminescence imaging of mice treated with vehicle control or different doses of linezolid (25 or 50 mg/kg)). Images were acquired at the first sign of neurological deterioration in any cohort. Right: Quantification of tumour flux in each group. N = 5 mice per group. b, Tumour growth curve from in vivo bioluminescence analysis of mice (n = 5 mice per group) treated with vehicle control or linezolid (25 or 50 mg/kg) after orthotopic GSC 23 tumour implantation. c, Kaplan–Meier survival curves of mice treated with vehicle control or linezolid (25 or 50 mg/kg) after orthotopic GSC 23 tumour implantation. N = 5 mice per group. d, Left: Representative in vivo bioluminescence imaging of mice treated with vehicle control or linezolid (25 or 50 mg/kg). Images were acquired at the first sign of neurological deterioration in any cohort. Right: Quantification of tumour flux in each group. N = 5 mice per group. e, Tumour growth curve from in vivo bioluminescence analysis of mice treated with vehicle control or linezolid (25 or 50 mg/kg) after orthotopic GSC 3565 tumour implantation. N = 5 mice per group. f, Kaplan–Meier survival curves of mice treated with vehicle control or linezolid (25 or 50 mg/kg) after orthotopic GSC 3565 tumour implantation. N = 5 mice per group. g, Tumour growth curve from in vivo bioluminescence analysis of GSC23 tumour-bearing mice treated with vehicle control, linezolid (50 mg/kg), meclofenamate (Mec, 20 mg/kg), or linezolid and meclofenamate in combination. N = 5 mice per group. Right: Kaplan–Meier survival curves of mice treated with vehicle control, linezolid (50 mg/kg), meclofenamate (20 mg/kg), or linezolid and meclofenamate in combination. N = 5 mice per group. h, Tumour growth curve from in vivo bioluminescence analysis of GSC 3565 tumour-bearing mice treated with vehicle control, linezolid (50 mg/kg), Meclofenamate (Mec, 20 mg/kg) as single agents and in combination. N = 5 mice per group. Right: Kaplan–Meier survival curves of mice treated with vehicle control, linezolid (50 mg/kg), meclofenamate (20 mg/kg), or linezolid and meclofenamate in combination. N = 5 mice per group. i, In vivo bioluminescence imaging of GSC23 tumour-bearing mice treated with vehicle control, temozolomide (TMZ, 20 mg/kg), linezolid (50 mg/kg), or temozolomide and linezolid in combination. N = 5 mice per group. j, Kaplan-Meier curves of GSC23 tumour-bearing mice treated with vehicle control, temozolomide (TMZ, 20 mg/kg), linezolid (50 mg/kg), or temozolomide and linezolid in combination. N = 5 mice per group. k, Left: Haematoxylin and eosin stain of GSC23 tumour-bearing mice treated with vehicle control, temozolomide (TMZ, 20 mg/kg), linezolid (50 mg/kg), or temozolomide and linezolid in combination. Middle: In vivo immunofluorescence slides revealing the expression patterns of SOX2 and Ki67 in tumour tissues post-treatment with single agents or combination therapy. Scale bar: 40 μm. Right: Quantitative analysis of SOX2^+^ and Ki67^+^ positive cells. N = 5 random fields from triplicate samples. Data are presented as mean ± SD. Two-way ANOVA followed by multiple comparisons with adjusted p-values for b and e. One-way ANOVA followed by multiple comparisons with adjusted p-values for a, d, and k. Log-rank test for c, f, g, h.

To determine if the effects of linezolid against tumours translated into preclinical therapeutic efficacy, we examined in vivo efficacy against GBMs. Mice orthotopically implanted with GSCs transduced with a bioluminescent reporter showed reduced tumour volumes when treated with linezolid (50 mg/kg) as compared to vehicle treatment (Fig. 7a-d), which translated into increased survival of tumour-bearing mice (Fig. 7e, f). We evaluated the effects of linezolid on tumour cell expression of glutamate receptors and cancer stem cell markers. Linezolid treatment reduced the expression of glutamate receptors (GluR1; Extended Data Fig. 7f) and stemness markers (SOX2; Extended Data Fig. 7g). These results establish linezolid as a potent therapeutic agent that compromises glioma network integrity to suppress glioma progression.

### Linezolid displays combinatorial efficacy with gap junction inhibition and chemotherapy

Efficacy of linezolid as monotherapy against tumour growth was promising, but potential benefit can be derived from therapeutic combinations with agents that have orthogonal modes of action. Previous pharmacologic strategies to target neuronal stimulation of tumour growth have often focused on disrupting the pre-synaptic, neuronal connections to tumour cells. Meclofenamate (Mec) is an FDA-approved, brain-penetrant drug that functions as both a COX inhibitor and potent blocker of gap junctions interposed within tumour microtubes (TMs), thereby disrupting TM-mediated tumour–tumour communication and attenuating network-driven glioma growth^3,4^. In contrast, linezolid targets the postsynaptic tumour cell connections with neurons. To assess whether simultaneous inhibition of TM-mediated tumour connectivity and postsynaptic signalling enhances th determine the potential in vivo benefit of this combinatorial strategy, we implanted GSCs into mice, allowed tumours to become established, and then separated tumour-bearing mice into four treatment arms: vehicle (DMSO) control, linezolid monotherapy, Mec monotherapy, or combination of linezolid and Mec. Each monotherapy showed anti-tumour activity with decreased tumour growth and increased survival compared to vehicle control, whereas combined linezolid and Mec treatment produced greater tumour suppression compared to either agent alone (Fig. 7g, h). Thus, combined targeting of TM-mediated tumour cell–tumour cell and neuron–tumour cell connectivity disrupts glioma network communication and suppresses tumour progression.

GSCs display relative resistance to conventional therapies, engendering efforts to identify therapies to induce greater sensitivity. Therefore, we next evaluated the therapeutic potential of linezolid in clinically relevant combination treatment strategies. The oral methylator temozolomide (TMZ) standard-of-care chemotherapy to treat GBM with GSCs relatively resistant to TMZ^57,58^. We measured effects of linezolid and TMZ on GSC sphere formation; each agent showed efficacy as monotherapy with the combination offering additional benefit (Extended Data Fig. 7i). A range of concentrations of linezolid and TMZ were tested for anti-proliferative activity against two patient-derived GSCs, supporting synergistic anti-tumoural efficacy (Extended Data Fig. 7g). Mice bearing orthotopic tumours generated from patient-derived GSCs were treated in one of four groups: vehicle control, TMZ monotherapy, linezolid monotherapy, or the combination of TMZ and linezolid. TMZ and linezolid showed independent activity as measured by reduced tumour volume measured by bioluminescence with greater efficacy in combination (Fig. 7i), which translated into prolonged survival (Fig. 7j) and decreased direct tumour size (Fig. 7k). These findings suggest that linezolid augments efficacy of TMZ as combinatorial therapy for GBM.

## DISCUSSION

The convergence of cancer biology and neuroscience has revealed new levels of complexity and therapeutic directions in brain tumours and other cancers. The connectivity between neurons and tumour cells, and among tumour cells themselves, enables the modelling of tumour growth beyond the limitations of isolated cell proliferation, thereby promising to inform improved therapeutic strategies. Optogenetic studies have delineated how neuronal activity drives tumour growth via synaptic inputs, and tumour cells also autonomously establish functional multicellular networks mediated by TMs to confer therapy resistance and promote malignant progression. However, the molecular governance of tumour cells driving the formation of synapses and growth of networks has been poorly understood. Here, we have focused on the structural elements, TMs, which are the physical entities within tumour cells associated with network formation and confer resistance to therapy and promote malignant progression^59^. To date, mechanisms underlying TM formation and their functions in GSCs remain unclear.

We found that stem-like tumour cells were enriched for axon-related gene expression and growth of TMs relative to differentiated tumour cells in vitro and in vivo. To dissect TM function in GSCs, we isolated TMs and conducted transcriptomic and proteomic analyses, identifying enrichment of translation and protein synthesis within TMs. In normal neurons, local protein synthesis enables rapid responses to environmental stimuli^43,60^ and promotes axonal maintenance, guidance, communication, and synaptic plasticity^39,61–64^. We found selective translation in neurite-like GSC TMs, reflected by AMPA receptor expression, observed with a fluorescent, laser activated reporter of glutamate translation and OPP incorporation. GSC TMs showed higher local protein synthesis than differentiated glioma cells (DGCs), neurons, and astrocytes. Pharmacological blockade of local translation impaired TM-mediated network communication, which we attribute to the depletion of locally synthesized connectivity proteins — including gap junction components and synaptic receptors — which require continuous replenishment to sustain intercellular signalling.

Proteomic and bioinformatic studies identified increased mitochondria protein levels and mitochondrial oxidative phosphorylation in GSC TMs, like proteomics from axons and synapses of neurons^65–67^. We observed that elongated mitochondria were enriched and exhibited active long-range trafficking within GSC TMs. These highly motile, fused mitochondria played a critical role in TM formation and network establishment by dynamically translocating to generate ATP for local protein synthesis and to facilitate Ca^2+^ signal propagation, thereby maintaining intracellular calcium homeostasis and regulating cell survival. Based on this observation, siRNA screening of mitochondrial-related genes identified FASTKD2 as an essential regulator of local protein synthesis in GSC TMs and TM formation. Knockdown of FASTKD2 decreased TM mediated intercellular communication in vitro and in vivo.

Mitochondrial RNA granules are centres for posttranscriptional RNA processing and biogenesis of mitochondrial ribosomes. FASTKD2 is an RNA binding protein and important component of mitochondrial RNA granules that regulates the mitochondrial 16S rRNA and intra-mitochondrial translation^52,68^. We found that FASTKD2 distributed and slid along mitochondria in GSC TMs more than in cell bodies and drove local protein synthesis in GSC TMs, sustaining ATP production in GSC TMs by increasing the mitochondrial respiratory chain expression to maintain the TCA cycle. FASTKD2 has been reported to be involved in the expression of the mitochondrially encoded respiratory complex proteins^69–71^, we analysed the FASTKD2 interactome to reveal an binding to YTHDF2 within mitochondrial RNA granules. The interaction between FASTKD2 and YTHDF2 likely contributes to mitochondrial RNA processing and/or transport in GSC TMs. Given that the length of TMs can extend up to 500 μm^72^ and that ATP produced within TMs serves as a critical energy source for the cell, maintaining steady energy supplies to the distal regions of TMs are likely particularly challenging for GSCs. Mitochondrial RNA granules possess fluid-like properties, enabling movement along the inner mitochondrial membrane, which supports continuous content exchange as well as promotes mitochondrial translation and oxidative phosphorylation^73^. Hyperfused elongated mitochondria have been observed in TMs, potentially offering more space for mitochondrial RNA granules and facilitating their transport to distal TM regions to support mitochondrial function.

We found that YTHDF2 and FASTKD2 bound to MT-RNR1, a mitochondrial ribosomal RNA, to promote its stability in GSC TMs. As FASTKD2 is a key protein within mitochondrial granules and its knockdown only affected MT-RNR1 expression in TMs, not the whole cell level, FASTKD2 appears to regulate transport of MT-RNR1 within the TM. YTHDF2 knockdown not only reduced MT-RNR1 levels but also decreased the binding between MT-RNR1 and FASTKD2 in TM, indicating that YTHDF2 may stabilize MT-RNR1 in TMs. We observed that the C-terminal domain of YTHDF2 was critical for its interaction with FASTKD2, aligning with previous reports of domain-specific binding^74^. However, whether this interaction is mediated by m^6^A recognition on MT-RNR1 remains unclear. Although YTHDF2 is well known for regulating m^6^A-dependent RNA stabilization and localization, a mechanism established in neurons for directing mRNAs to dendrites and axons^75^, its relevance to mitochondrial transcripts in GSC TMs has not been demonstrated. Additionally, we previously demonstrated that GSCs specifically express YTHDF2 to promote tumour growth^76^. Based on the current results, we propose that YTHDF2 plays distinct roles in axon-like tubular structures, differing from its function in other cell types or environments. Mitochondrial RNA granules are structurally similar to stress granules and P-bodies^73^, and YTHDF2 is involved in the formation of both^77,78^. Therefore, YTHDF2 may contribute to the formation of mitochondrial RNA granules through phase separation in TMs. Future studies will investigate YTHDF2 involvement in the formation or localization of mitochondrial RNA granules in GSC TMs. Collectively, FASTKD2 and YTHDF2 function collaboratively to facilitate the transport and stabilization of MT-RNR1, respectively, ensuring its efficient integration into mitochondria within GSC TMs. This partnership not only promotes the accumulation of mitochondrial RNA granules but also supports energy production required for localized protein synthesis, thereby enabling GSCs to maintain their growth, communication, and survival.

Glioma cells enhance intercellular communication by utilizing neuronal developmental mechanisms, which facilitates tumour progression. In neuronal axons, local translation within axons provides rapid and spatially confined sources of synaptic proteins that shape excitability and circuit plasticity. ^42^ In GBMs, TMs not only promote glioma cell intercellular communication but also facilitate the transmission of neuronal signals^5^. We found that mitochondria within GSC TMs provide the energetic and biosynthetic support required for TM formation and local synthesis of neuronal receptors, thereby enhancing intercellular communication. FASTKD2 regulation of local translation supports intercellular communication in GSC TMs. Further studies will probe the downstream effects and implications of the functional electrical calcium signalling ongoing between GSC TMs and neurons.

To translate these findings into a therapeutic paradigm, we focused on drug repositioning to target local protein synthesis in GSCs by screening an FDA-approved drug library. The orally available antibiotic linezolid was identified as a potential candidate targeting FASTKD2. Linezolid binds to a site on the bacterial 23S ribosomal RNA of the 50S subunit, preventing the formation of a functional 70S initiation complex^79^. Linezolid crosses the blood-brain barrier and is used in central nervous system infections^80,81^, suggesting that it could be directly translated into clinical trial for brain tumours. Linezolid treatment inhibited mitochondrial function and ATP production in GSCs with reduction in the initiation of local protein synthesis and intercellular communication. Consequently, we found linezolid to have anti-tumour effects in mice xenografted with orthotopic brain tumours. When combined with TMZ, linezolid displayed combinatorial benefit, further extending the survival of tumour-bearing mice compared to monotherapy alone. As linezolid frequently induces thrombocytopenia^82^, like TMZ, dose finding studies for patients would be required to avoid increased toxicity.

In summary, we demonstrate that GSCs and neurons share structural features, specifically TMs, which phenocopy neuronal cell biology. TMs harbour local protein synthesis in GSCs and promote glioma intercellular communication and GSC survival. We identified FASTKD2 as a clinically relevant gene that drives tubule formation and facilitates local protein synthesis in GSCs by regulating mitochondrial function. Linezolid inhibits FASTKD2 and displays anti-tumour effects in vitro and in vivo by disrupting TM formation and cell-cell communication. Collectively, local protein synthesis provides another example of lineage plasticity in cancer and informs potential therapeutic targeting for GBM.

### RESOURCE AVAILABILITY

#### Data and code availability

RNA-seq data for three pairs of GSCs and DGCs are from GSE54791. RNA-seq data for 44 GSC and 6 NSC lines are from GSE41031. Matched pairs of GSCs and DGCs H3K27ac ChIP-seq data are from GSE129438. GSCs and NSCs H3K27ac ChIP-seq are from GSE119755. scRNA-seq and snRNA-seq data are publicly available in Broad Institute Single-Cell Portal (https://singlecell.broadinstitute.org/single_cell/study/SCP503). The code supporting the current study is available from the lead contact upon request. This study does not generate new code, algorithm or software. Any additional information required to reanalyse data is available from the lead contact upon request.

### MATERIALS AND METHODS

#### GSC, NSC, and DGC derivation and maintenance

GBM tissues were obtained from excess surgical resection samples from patients at Case Western Reserve University after neuropathological review and diagnosis. Written informed consent was obtained from all patients under the protocol approved by the Institutional Review Board (IRB# 090401). All procedures were conducted in accordance with the principles of the Declaration of Helsinki and relevant institutional guidelines. To minimize in vitro cell culture-based artifacts, patient-derived xenografts (PDXs) were propagated as a renewable source of glioblastoma stem-like cells (GSCs). GSC23 was derived from a recurrent GBM biopsy sample from a 63-year-old male patient and was provided by E. Sulman. GSC3565 was derived in our laboratory and transferred under a material transfer agreement (MTA) from Duke University. HNP1 human neural progenitors (ArunA Biomedical) were fully differentiated and derived as adherent cells from the human embryonic stem cell (hESC) WA09 line. The human neural stem cell (NSC) line NSC11 (ALSTEM) was derived from human induced pluripotent stem cells (hiPSCs). ENSA (ENStem-A, Millipore) are human embryonic stem-derived neural progenitors. All GSC and NSC lines were maintained in Neurobasal medium (Gibco, Cat# 21103049) supplemented with 0.8 mM L-lysine (Sigma-Aldrich, Cat# L5501), B27 supplement without vitamin A (Gibco, Cat# 12587010), 20 ng/ml epidermal growth factor (EGF) (R&D Systems, Cat# 236-EG-200), 20 ng/ml recombinant human basic fibroblast growth factor (bFGF) (R&D Systems, Cat# 233-FB-025), 1 mM sodium pyruvate (Gibco, Cat# 11360070), 2 mM GlutaMAX (Gibco, Cat# 35050061), and 100 U/ml penicillin/100 μg/ml streptomycin (Gibco, Cat# 15140122). Cells were incubated at 37°C in a humidified atmosphere with 5% CO_2_. Differentiated glioma cells (DGCs) were maintained in Dulbecco’s modified Eagle’s medium (DMEM) (Gibco, Cat# 11965092) supplemented with 10% heat-inactivated foetal bovine serum (FBS) to sustain differentiation status.

#### Cell Culture and lentiviral transduction

293T was purchased from American Type Culture Collection and cultured in DMEM supplemented with 10% foetal bovine serum (cat: 26140079; Gibco) and streptomycin/penicillin (cat: 15140122; Gibco) at 37°C with 5% CO2. Lentiviral clones expressing two non-overlapping shRNAs directed against FASTKD2 or a non-targeting control shRNA encoding a sequence does not present in the mammalian genome (shCONT) were obtained from Sigma-Aldrich. Lentiviral overexpression plasmid for FASTKD2, YTHDF2 and MT-RNR1 and empty vector were ordered from VectorBuilder Inc. 293T cells were used to generate lentiviral particles through co-transfection of the packaging vectors pCMV-dR8.2 dvpr and pCI-VSVG. Cells were transfected with lentivirus for 24 h, followed by recovery for 24 h before selection. Infected cells were selected with 2 mg/ml puromycin until uninfected control cells were dead. All cell lines in this study were tested to confirm lack of mycoplasma contamination. The genomic identity of each cell line has been authenticated by Duke University DAN Analysis Facility. The shRNA sequences are listed in Supplementary Table 6.

#### Xenografts

Murine studies were conducted in full compliance with the guidelines of the University of Pittsburgh’s Institutional Animal Care and Use Committee (Protocol number 21049014). Male and female mice were kept under controlled conditions, including a specific-pathogen-free environment, temperature maintained between 20–26°C, humidity of 30–70%, and a consistent 12-hour light-dark cycle. In mice experiments, the maximum tumour size permitted was 15 mm in any direction. For intracranial xenografts using human-derived GSCs, and tumour cells were infected with lentiviruses encoding GFP, GCaMP6f, shCONT, or shFASTKD2 prior to implantation, NOD.Cg-*Prkdc*^*scid*^ *Il2rg*^*tm1Wjl*^/SzJ (NSG) mice aged between 5–6 weeks were selected randomly. These mice underwent intracranial injection. Approximately 100,000 cells in 2 μl sterile PBS were stereotactically implanted into the CA1 region of the hippocampus through a 31-gauge burr hole at a depth of 3 mm, adhering to standard protocols, After drug treatment (TMZ, 20mg/kg; MedChemExpress; formulated in 10% DMSO in PBS), Linezolid (50mg/Kg; MedChemExpress; formulated in 10% DMSO in PBS), meclofenamate sodium (20 mg/kg; MedChemExpress; formulated in 10% DMSO in PBS) or control 10% DMSO). Injections were administered every other day for a total of 3 weeks. mice were closely observed for any neurological symptoms and humanely euthanized once euthanasia criteria were met. Mice brains were extracted, fixed in 4% paraformaldehyde (PFA), cryopreserved in 30% sucrose, and subsequently cryosectioned for analysis. Tissue sections underwent haematoxylin and eosin staining for histological examination. Survival rates of the mice were statistically analysed using GraphPad Prism software, employing log-rank tests to determine significance. Additionally, bioluminescence imaging was conducted for mice implanted with GSCs tagged with firefly luciferase. This involved treating the animals with D-luciferin (50 mg/kg), followed by anaesthesia with isoflurane to facilitate the imaging process. The bioluminescence images were captured using PerkinElmer’s IVIS imaging system.

#### Proliferation assays

Cell proliferation experiments were conducted by plating 2,000 cells per well in 96-well plates with three technical replicates per condition. Cell viability was measured using CellTiter-Glo (Promega, Cat# G7570) and data were presented as mean ± SD.

#### Culture in microfluidic chambers

Microfluidic chips (Xona Microfluidics) were pre-coated with poly-D-lysine (Sigma-Aldrich) and laminin (Sigma-Aldrich), then placed into 10 cm cell culture dishes (Corning) containing 2–3 mL of PBS (Gibco) to maintain humidity. A total of 1 × 10^5^ cells were seeded in complete Neurobasal medium (Gibco) into one side of each chamber. After 72 hours, these cultures were used for subsequent immunofluorescence and drug treatment experiments. For drug treatment, 150 μL of the drug solution or DMSO (Sigma-Aldrich) was added to the treatment side, while 200 μL of fresh culture medium was placed on the non-treatment (control) side to maintain optimal hydrostatic pressure. Cells were incubated under these conditions as indicated before further analysis.

#### TM isolation

TM isolation from cell culture followed previously reported axon isolation procedures^83^ with minor modifications. Briefly, transwell filter inserts containing a polyethylene terephthalate (PET) membrane with a 3 μm pore size and 24 mm diameter were precoated with poly-L-lysine and laminin. One million GSCs were then plated. To evaluate axonal growth through the membrane, inserts were rinsed in PBS after 48 hours of cell culture and processed for immunofluorescence. To isolate GSC TMs, the top membrane surface of each insert was scraped with a cotton-tipped applicator. Scraping was repeated three times with a fresh applicator and performed in different directions. To isolate GSC cell bodies, the surface underneath the membrane was scraped in an identical manner as above. Scraped membrane lysates were subsequently used for protein extraction, RNA extraction, and further biochemical and molecular analyses.

#### Mitochondrial isolation

Cell body or GSC TMs were resuspended in ice-cold 250 mM sucrose/10 mM Tris-HCl (pH 7.4) and homogenized with seven passes in a pre-chilled, zero-clearance homogenizer (Kimble/Kontes). A post-nuclear supernatant was obtained by centrifugation of the samples twice for 10 minutes at 600 × g. Mitochondria were pelleted by centrifugation for 10 minutes at 10,000 × g and washed once. Protein concentration was determined by bicinchoninic acid (BCA) assay.

#### Glioma–neuronal co-culture

The GSC-neuron in vitro co-culture used in this study has previously been described^1^.Briefly, GSCs were plated on poly-D-lysine- (P6407, Sigma-Aldrich) and laminin-coated (L2020, Sigma-Aldrich) coverslips (Neuvitro) at a density of 10,000 cells per well in 24-well plates (CLS3524, Corning). After 24 hours, 40,000 embryonic mouse hippocampal neurons (Gibco, A15586–01) were seeded onto the glioma cells (FASTKD2 knockdown and shCONT) and maintained in serum-free Neurobasal medium (21103049, Gibco) supplemented with B27 (17504044, Gibco), gentamicin (15750060, Gibco), and GlutaMAX (35050061, Gibco). Co-cultures were maintained for one week before fixation with 4% paraformaldehyde (PFA, 157–8, Electron Microscopy Sciences) for subsequent assays. For microfluidic chamber experiments, glioma cells were cultured for several days before neurons were seeded onto the opposite side of the chamber, just before the transition medium was introduced through the channel. Subsequent treatments, including drug administration or transient gene knockdown, were applied to the glioma cell side of the chamber.

#### EdU incorporation assay

Coverslips were precoated with poly-D-lysine (P6407, Sigma-Aldrich) and laminin (L2020, Sigma-Aldrich) prior to cell seeding. After neuron culture for five days, glioma cells with FASTKD2 knockdown or shCONT were added to the neuronal cultures on the slides. To evaluate the effect of linezolid on neuronal regulation of glioma cell proliferation, 100 μM linezolid or DMSO (vehicle control) was added to the co-culture wells. Cells were co-cultured with or without linezolid for 48 hours, followed by incubation with 10 μM EdU (C10337, Invitrogen) for 2 hours. Subsequently, cells were fixed with 4% paraformaldehyde (PFA, 157–8, Electron Microscopy Sciences). For EdU detection, cells were processed using the Click-iT EdU Imaging Kit (C10337, Invitrogen) according to the manufacturer’s instructions. The proliferation index was determined by quantifying the percentage of EdU-labelled glioma cells (EdU^+^/DAPI) relative to the total number of glioma cells using confocal microscopy (LSM 880, Zeiss).

#### Proximity ligation assay (PLA)

The proximity ligation assay was performed using the Duolink Proximity Ligation Assay Kit (DUO92101, Sigma-Aldrich). Briefly, fixed glioma cells were incubated with primary antibodies (FASTKD2, anti-puromycin, or YTHDF2) diluted in Duolink antibody diluent at 4°C overnight, then washed three times with Buffer A (0.01 M Tris, 0.15 M NaCl, 0.05% Tween 20) at RT for 5 minutes each, incubated with Duolink PLA Probe at 37°C for 1 hour, rewashed with PBS three times at RT for 5 minutes each, then incubated in Duolink PLA ligation solution at 37°C for 30 minutes, rewashed with PBS three times at RT for 5 minutes each, then incubated with Duolink PLA amplification solution (DUO92009, Sigma-Aldrich) at 37°C for 100 minutes, washed with 1x Buffer B (0.2 M Tris, 0.1 M NaCl pH 7.5) two times at RT for 10 minutes each, and finally, PBS at RT for 5 minutes. To counterstain for a neuronal marker, neurons were post-fixed with 4% paraformaldehyde and 4% sucrose for 10 minutes at RT and processed for immunocytochemistry.

#### Immunofluorescence staining and imaging

Cells were grown in microfluidic devices with precoated poly-d-lysine and laminin coverslips. Next, coverslips were fixed in 4% paraformaldehyde (PFA) and 120 mM sucrose for 20 minutes and washed three times with PBS. Cells were permeabilized in 0.25% Triton X-100 in PBS for 10 minutes and washed once with PBS. Then, cells were blocked in PBS containing 0.1% (v/v) Triton X-100 and 10% (w/v) bovine serum albumin (BSA) for 1 hour. Primary antibodies were diluted in PBS containing 0.1% (v/v) Triton X-100 and 1.0% (w/v) BSA and added to cells at 4 °C overnight. Primary antibodies: YTHDF2 (Rabbit, NOVUS, Cat# NBP3–25763, 1:500, RRID: AB_3635893), FASTKD2 (Rabbit, NOVUS, Cat# NBP1–55360, 1:1000, RRID: AB_11030882), SOX2 (Goat, NOVUS, Cat# AF2018, 1:500, RRID: AB_355110), YTHDF3 (Rabbit, NOVUS, Cat# NBP3–25764, 1:500, RRID: AB_3635894), METTL3 (Rabbit, NOVUS, Cat# NBP3–22009, 1:500, RRID: AB_3622715), P53 (Rabbit, NOVUS, Cat# NB200–103, 1:1000, RRID: AB_10001083), PSD95 (Mouse, NOVUS, Cat# NB300–556, 1:1000, RRID: AB_2092366), SYNAPSIN-1 (Rabbit, NOVUS, Cat# NB300–104, 1:1000, RRID: AB_10078308), GLUR1 (Rabbit, NOVUS, Cat# NBP2–22399, 1:1000, RRID: AB_2857850), TOMM20 (Mouse, NOVUS, Cat# H00009804-M01, 1:1000, RRID: AB_519121), Ki67 (Mouse, NOVUS, Cat# NBP2–22112, 1:500, RRID: AB_3266719), NESTIN (Chicken Polyclonal, NOVUS, Cat# NB100–1604, 1:500, RRID: AB_525740), NESTIN (Mouse, NOVUS, Cat# MAB1259, 1:500, RRID: AB_525740), NF-L (Rabbit, NOVUS, Cat# NB300–131, 1:1000, RRID: AB_2251212), EGFR (Mouse, Cell Signaling Technology, Cat#66278, RRID: AB_28595),. Cells were then washed with PBS and incubated with appropriate secondary antibodies (1:1000 dilution) for 1 hour at RT. secondary antibodies: Alexa Fluor 647 Donkey anti-Mouse IgG (Donkey, Life Technologies, Cat# A20186, 1:500, RRID: AB_2536183),Alexa Fluor 488 Donkey anti-Rabbit IgG (Donkey, Life Technologies, Cat# A10037, 1:500, RRID: AB_11180865),Alexa Fluor 568 Donkey anti-Rabbit IgG (Donkey, Life Technologies, Cat# A10042, 1:500, RRID: AB_2534017), Alexa Fluor 488 Donkey anti-Chicken IgG (Donkey, Life Technologies, Cat# A78948, 1:500, RRID: AB_2921070),Alexa Fluor 555 Donkey anti-Goat IgG (Donkey, Life Technologies, Cat# A-11055, 1:500, RRID: AB_2534102).After washing, nuclei were stained with DAPI solution for 3 minutes at RT. After two washes in PBS, cells on microfluidic devices were imaged directly, while coverslips were mounted on glass slides (Globe Scientific) and allowed to dry at RT overnight. Confocal images were obtained on a Lecia SP8 confocal microscope.

#### siRNA transfection

siRNA was obtained from RiboBio. siRNA was mixed with transfection reagent lipofectamine RNAiMAX (Invitrogen) diluted in Opti-MEM (Gibco) and incubating for 20 minutes before addition to cells. siRNA transfection conditions were optimized for each cell line. For transfection, 96-well plates were precoated with Matrigel, and GSC cells were seeded at 500 cells/well and cultured for 72 hours. Next, 30 nm of siRNA in a final volume of 100 μL were added to each well. After 6 hours, the neurobasal culture medium was changed, and cells were cultured for an additional 72 hours. The supernatant was then removed, and a fresh medium containing puromycin (10 μM) was added. Cells were then incubated for 30 minutes, washed with PBS three times, and fixed for 15 minutes using 4% paraformaldehyde and processed for further experiments. The siRNA sequences are listed in Supplementary Table 6.

#### siRNA screening

After siRNA transfection, fixed cells were washed in PBS and permeabilized (0.5% Triton^®^ X-100 in PBS) for 10 minutes. The Click-iT^®^ Plus OPP Protein Synthesis Assay Kit (Thermo Fisher Scientific) was used to detect protein synthesis as described in the protocol^84^. Fluorescence data from the Opp-647 screen were compared to the negative control per plate, Robust Z-scores were calculated based on the number of OPP puncta within the terminal 10 μm segment of each TM. Tubular structures (TMs) were visualized by staining GSCs with Alexa Fluor 488-conjugated phalloidin (1:1000 dilution), washing 3 times with PBS, and imaging using 20x Magnification on a Leica SP8 confocal microscope. Images were processed using ImageJ. TM length was measured by Neuron J, and TM length were compared to the negative control per plate to calculate robust Z-scores. For high throughput flow cytometry, a 96-well plate was precoated with poly-D-lysine and laminin and GSCs were seeded with a cyto-ATP reporter. After siRNA library transfection for 72 hours, cells were digested into single cells, and ATP intensity was detected using high throughput flow cytometry. High-throughput screening data was compared to control per plate, and robust Z-scores were calculated using the formula z = (X − μ)/s.d. where μ is the mean of the negative controls and s.d. is the standard deviation of the whole population. X is the sample value calculated based on the integrated fluorescent intensity/ number /length of the indicators.

#### Western blotting

Cells were collected and lysed in RIPA buffer (50 mmol/L Tris-HCl, pH 7.5; 150 mmol/L NaCl; 0.5% NP-40; and 50 mmol/L NaF with protease inhibitors) and incubated on ice for 1 hour. Lysates were centrifuged at 4°C for 10 minutes at 12,000 rpm, and the supernatant was collected. Equal amounts of protein samples were mixed with SDS Laemmli loading buffer, boiled for 5 minutes at 100°C, electrophoresed using NuPAGE Bis-Tris gels, and transferred onto nitrocellulose membranes. 5% nonfat dry milk in TBST was used for blocking for 1 hour followed by primary antibody incubation (1:1000 dilution) at 4°C overnight. primary antibody: DHA (Rabbit, Cell Signaling Technology, Cat# 5839, 1:1000, RRID: AB_10707493), SOX2 (Rabbit, Cell Signaling Technology, Cat# 23064, 1:500, RRID: AB_2714146), GFAP (Rabbit, Cell Signaling Technology, Cat# 3670, 1:1000, RRID: AB_561049), OLIG2 (Rabbit, Cell Signaling Technology, Cat# 65915, 1:500, RRID: AB_2936997), YTHDF2 (Rabbit, Cell Signaling Technology, Cat# 80014, 1:500, RRID: AB_2923056), MTCO2 (Rabbit, Proteintech, Cat# 55070–1-AP, 1:1000, RRID: AB_2934972), β-ACTIN (Rabbit, Proteintech, Cat# 81115–1-RR, 1:5000, RRID: AB_2923704), FASTKD2 (Rabbit, Proteintech, Cat# 17464–1-AP, 1:1000, RRID: AB_2101119), ATP6 (Rabbit, Proteintech, Cat# 55313–1-AP, 1:1000, RRID: AB_2881305), ATP8 (Rabbit, Proteintech, Cat# 26723–1-AP, 1:1000, RRID: AB_2880614), HSP90 (Rabbit, Proteintech, Cat# 13171–1-AP, 1:1000, RRID: AB_2120924), ND1 (Rabbit, Proteintech, Cat# 19703–1-AP, 1:1000, RRID: AB_10637853), ND2 (Rabbit, Proteintech, Cat# 19704–1-AP, 1:1000, RRID: AB_10638920), NDUFV1 (Rabbit, Proteintech, Cat# 11238–1-AP, 1:1000, RRID: AB_2149040), UQCRC2 (Rabbit, Proteintech, Cat# 14742–1-AP, 1:1000, RRID: AB_2934901), YTHDF2 (Rabbit, Proteintech, Cat# 24744–1-AP, 1:500, RRID: AB_2919873), GAP43 (Rabbit, NOVUS, Cat# NB300–143, 1:1000, RRID: AB_10001196), VDAC1 (Rabbit, NOVUS, Cat# NB100–695, 1:1000, RRID: AB_10000851). Following overnight primary antibody incubation, membranes were washed three times (5 minutes each) with TBST. Finally, membranes were incubated with secondary antibody (Anti-rabbit IgG, HRP-linked (Goat, Cell Signaling Technology, Cat# 7074, 1:2000, RRID: AB_2099233), Anti-mouse IgG, HRP-linked (Goat, Cell Signaling Technology, Cat# 7076, 1:2000, RRID: AB_330924)for 1 hour at RT, washed with TBST, and imaged using the Bio-Rad Image System.

#### Cellular thermal shift assay

GSC cells were plated in 100 mm culture dishes at a density of 1×10^7^ cells. Next, cells were treated with either linezolid or DMSO for a duration of 4–8 hours. Following treatment, cells were washed with PBS containing protease inhibitors, resuspended in the same PBS with inhibitors, and centrifuged at 1200 rpm for 5 minutes. The pellet was resuspended in 500 μL of PBS with protease inhibitors and 27 μL portions of were transferred into PCR tubes. These cells underwent treatment in a thermocycler in which the temperature incrementally rose from 40 to 72 °C for 3 minutes. Following treatment, cells were allowed to equilibrate to room temperature for 3 minutes before quick-freezing in liquid nitrogen. Cells were then lysed by cycling between freezing in liquid nitrogen and thawing in a water bath at 25°C; this process was repeated three times. The lysates were then centrifuged at 12,000 × g for 10 minutes to pellet the debris. The clear supernatant was decanted and mixed with 4X Laemmli loading buffer and boiled for 5 minutes at 100°C. Finally, prepared samples were analysed via western blotting.

#### Live imaging

For local translation assays, the Dendra2 fluorescence protein was conjugated with UTRs of GluR1 to create a 5′UTR-GluR1-Dendra2–3′UTR reporter (Vectorbuilder). GSCs were cultured on microfluidic devices (Xona Microfluidics) for 3 days and then transfected with this reporter. 24 hours following transfection, the following protein synthesis assay was performed: Existing fluorescence of Dendra2 (green) in the axonal tip was photoconverted into red fluorescence with a 405 nm laser for 10 seconds, and newly synthesized Dendra2 (green) signals were measured for 20 minutes with time-lapse imaging performed every 5 minutes. Images were acquired using 488 and 561 nm lasers.

#### Preparation of Coronal Slices and Electrophysiological Recording

Methods for brain slices and electrophysiological recording as reported before^85^. Brain slices (300 μm thick, Leica VT1200s) containing the hippocampal region were prepared from mice four weeks after xenografting. Following rapid decapitation, the brain was carefully removed from the skull and immediately immersed in ice-cold slicing artificial cerebrospinal fluid (ACSF) with the following composition: 125 mM NaCl, 2.5 mM KCl, 25 mM glucose, 25 mM NaHCO_3_, 1.25 mM NaH_2_PO_4_, 3 mM MgCl_2_, and 0.1 mM CaCl_2_. The slices were cut using a vibratome and then incubated for 30 minutes in warm (30°C), oxygenated (95% O_2_, 5% CO_2_) recovery ACSF containing: 100 mM NaCl, 2.5 mM KCl, 25 mM glucose, 25 mM NaHCO_3_, 1.25 mM NaH_2_PO_4_, 30 mM sucrose, 2 mM MgCl_2_, and 1 mM CaCl_2_. After incubation, the slices were allowed to equilibrate at room temperature for an additional 30 minutes. For electrophysiological recordings, the slices were transferred to a recording chamber and continuously perfused with oxygenated, warmed (28–30°C) recording ACSF containing: 125 mM NaCl, 2.5 mM KCl, 25 mM glucose, 25 mM NaHCO_3_, 1.25 mM NaH_2_PO_4_, 1 mM MgCl_2_, and 2 mM CaCl_2_. The slices were visualized using a microscope (Olympus BX61WI). Recording pipettes were generated from borosilicate glass (Flaming-Brown puller, Sutter Instruments, P-97, tip resistance of 2–3 MΩ) and were filled with pipette solution consisting of 130 mM potassium gluconate, 20 mM KCl, 5 mM sodium phosphocreatine, 10 mM HEPES, 4 mM Mg-ATP, 0.3 mM GTP, and 50 μM Fluo-4, pH = 7.3. Glutamate (1 mM; Sigma) dissolved in recording ACSF was applied for 250 ms via a puff pipette positioned approximately 100 μm away from the patched cell. The puff application was controlled using a Picospritzer II (Parker Hannifin). To prevent neuronal action potential firing during glutamate puff experiments, tetrodotoxin (TTX; 0.5 μM; Tocris) was included in the recording ACSF. Signals were acquired with a MultiClamp 700B amplifier (Molecular Devices) and digitized at 10 kHz with an Axon Digidata 1550B (Molecular Devices). Data were recorded and analysed using pClamp 11 software suite (Molecular Devices), IGOR Pro 8 (Wavemetrics).

#### In vivo calcium imaging

The genetically encoded pHAGE-RSV-tdTomato-2A-GCaMP6f (Addgene) was transduced via lentivirus into GSCs to monitor calcium activity. Calcium imaging was repeatedly performed in identical tumour regions four weeks after injection of Ca^2+^ sensor-expressing tumour cells into mice. Glioma cells were implanted into the dorsal hippocampus with shFASTKD2 or shCONT. At four weeks post-implantation, acute slices were prepared. In brief, brains were harvested and placed in ice cold cutting solution (92 mM N-methyl-D-glutamine, 2.5 mM KCl, 1.2 mM NaH2PO_4_, 30 mM NaHCO_3_, 20 mM HEPES, 25 mM glucose, 5 mM sodium L-ascorbate, 2 mM 878 thiourea, 3 mM sodium pyruvate, 10 mM MgSO_4_ and 0.5 mM CaCl_2_) and continuously bubbled with 95% O_2_ and 5% CO_2_. 200 μm-thick coronal sections were cut with a vibratome (Leica VT 1200S) and placed in artificial CSF, ACSF (126 mM NaCl, 2.5 mM KCl, 1.2 mM MgSO_4_, 2.4 mM CaCl_2_, 25 mM NaHCO_3_, 881 1.4 mM NaH2PO4, 11 mM glucose and 0.6 mM sodium L-ascorbate) and continuously bubbled with 95% O_2_ and 5% CO_2_. Slices were incubated at 31°C for 30 minutes and then at room temperature for 30 minutes. Live Ca^2+^ imaging was performed with a confocal microscope (Nikon AIR HD) on a 20X objective by acquiring images at 40 Hz in the 480 nm wavelength channel. Experiments were performed in a manner similar to that in a previously described report^13^. Simultaneous Ca^2+^ imaging was performed in the oxygenated ACSF. Ca^2+^ imaging recordings were quantified on ImageJ/FIJI. In brief, regions of interest (ROIs) of tdTomato were defined manually for each glutamate-responding GCaMP6f-expressing glioma cell using ImageJ. Mean intensity over the image time course was used to measure the corresponding change in fluorescence and following background subtraction, the intensity values were calculated as ΔF/F, where F is the basal fluorescence of the ROI and ΔF is the change in fluorescence of the ROI at peak response relative to the F value.

#### General image processing

Confocal micrographs were acquired using ZEISS ZEN software (Black edition 2012) and processed in Fiji (ImageJ2 2.3.0) for high-resolution 3D visualization and rendering. Data were visualized using single-plane views and maximum intensity projections with ‘Fire’ pseudocolor lookup tables (LUTs) to enhance dynamic range. TMs were strictly defined based on established morphological criteria: cellular protrusions exceeding 10 μm in length with a calibre between 0.5 and 2.5 μm. To quantify network topology, TMs and intercellular connections were manually assessed by two independent investigators; a functional connection was defined as physical continuity established by a TM bridging two distinct cell bodies or intersecting another TM.

#### Calcium imaging–based functional network analysis in cultured cells

Raw calcium imaging data were imported into Fiji (ImageJ2 2.3.0) for motion correction and region of interest (ROI) selection. Single-cell fluorescence trajectories were extracted and exported for downstream computational analysis. Graph theoretical analysis of Ca^2+^ activity was performed in MATLAB (R2020b, MathWorks Inc.) using custom scripts, essentially as previously described, with minor modifications. Briefly, single cell fluorescence traces were extracted from time lapse Ca^2+^ imaging movies, and only “active” cells, defined as traces containing at least four Ca^2+^ peaks, were included in the network analysis.

To quantify the strength of functional co-activity, we computed time-lagged Pearson correlation coefficients for all active cell pairs over 10-min recording intervals. Cross-correlation analysis was performed by temporally shifting traces relative to one another to account for signal propagation delays; the maximum correlation coefficient across all lags was defined as the connectivity strength. Signal propagation velocity was subsequently derived from the time lag at maximal correlation and the Euclidean distance between cell centroids. To exclude non-physiological artifacts, cell pairs were discarded based on the following criteria: (1) intercellular distance >100 μm; (2) estimated propagation speed outside the range of 4–25 μm/s; or (3) insufficient activity (<4 peaks).

To determine a significance threshold for coactivity while preserving the temporal autocorrelation of each trace, we generated a linear-shift null model, following published procedures. A significance threshold for functional co-activity was rigorously established using a linear-shift null model, designed to preserve the intrinsic temporal autocorrelation of individual traces while abolishing biological coupling. For each cell pair, one trace was randomly time-shifted by an interval of Delta T > 5 min relative to the other. This procedure generated surrogate datasets devoid of genuine synchronization. The 95th percentile of the resulting null distribution of maximal correlation coefficients was identified as r = 0.15 for our cultured GSC networks; consequently, only cell pairs exhibiting a correlation coefficient of 0.15 were classified as significantly co-active.

Undirected functional networks were constructed for each imaging field, where nodes represented individual active cells and edges denoted significant co-activity. Cells maintaining at least one functional edge (degree ≥1) were classified as ‘communicating cells,’ and their prevalence was quantified as a percentage of the total active cell population. To visualize the functional topology, network maps were spatially plotted using the x–y coordinates of cell centroids, distinguishing functional subpopulations including ‘periodic cells’ (defined by oscillation profiles) and ‘network hubs’—strictly defined as nodes within the top 5% of the degree distribution.

#### Electroporation of GSC23 cells

Electroporation was performed using a Bio-Rad Gene Pulser Xcell system based on previously reported protocols [Ref], with minor modifications. Briefly, about 2–3 million GSC23 cells grown as adherent tumour spheres in dish were enzymatically dissociated using Accutase, collected by gentle centrifugation (200 × g, 5 min), and resuspended in 900 μL of pre-warmed low-conductivity electroporation buffer (Opti-MEM with GlutaMAX). Aliquots of 100 μL (containing approximately 0.3 × 10^6^ cells) were mixed with the Cy3-UTP (0.5 mM). The mixture was transferred to 1 mm gap electroporation cuvettes (Bio-Rad #165–2088). A Bio-Rad Gene Pulser Xcell system was used in square-wave mode to deliver the pulse. Voltage, pulse length, pulse number and interval were set as programmable parameters. For GSC23, the starting conditions were 30–40 V (1 mm gap, ≈ 0.3–0.4 kV/cm) with a single pulse of 8–10 ms; these values can be fine-tuned (± 5 V or ± 2 ms) depending on cell viability and delivery efficiency. Typically, a voltage drop of 2–3 % of the set value was observed during the pulse.

After electroporation, the cell suspension was immediately transferred into one well of a 6-well plate containing 1 mL of 37 °C pre-warmed complete medium (Neurobasal + B27 + EGF/FGF2) and allowed to recover for 15–30 min before medium change. All procedures were performed at room temperature to maintain membrane fluidity. 24 hours post-electroporation, cells were examined for viability (Calcein-AM staining) and Cy3 fluorescence under a confocal microscope.

#### Measurement of the protein synthesis rate in vitro and in vivo

To evaluate protein synthesis in vitro, GSCs were cultured in complete neurobasal medium, and 2000 cells were plated in 12-well plates containing glass coverslips pre-coated with poly-d-lysine and laminin. After 72 hours, 10 mM Opp was added to the medium for 30 minutes. Cells were then fixed for 15 minutes by using 4% paraformaldehyde and processed as described in a prior report^84^. Briefly, fixed cells were washed in PBS, and permeabilized in a blocking buffer containing 0.05% saponin for 10 min. Next, the Click-iT^®^ Plus OPP Protein Synthesis Assay Kit (Thermo Fisher Scientific) was used to detect protein synthesis. To measure protein synthesis of GSC-derived xenografts in vivo, NSG mice were intracranially inoculated with GSCs in the right hemisphere until the study endpoint. A total of 20 μL of 1.5 mM O-propargyl-puromycin was injected into the contralateral hemisphere on day 28 using the following coordinates, zeroed at the bregma: −0.5 mm, 1.2 mm, −2 mm. Mice were euthanized 30 minutes after injection. Mice brains were collected, processed for cell digestion, labelled with antibodies, and underwent the Click-iT reaction using the Click-iT^®^ Plus OPP Protein Synthesis Assay Kit (Thermo Fisher Scientific). In vivo protein synthesis was based on the fluorescence intensity of the Alexa Fluor 647-OP-puro in labelled human tumour cells.

#### Mito-KillerRed evaluation

Mito-KillerRed is a photosensitizer that generates reactive oxygen species within mitochondria upon green light activation (excitation: 561 nm-emission: 601 nm). Prior to imaging, cells expressing Mito-KillerRed were protected from light to prevent inadvertent ROS production. Mito-KillerRed photostimulation (photobleaching) was performed by scanning the cells 20 times in 0.5 second intervals with a 561 nm laser at 100% intensity. The total time of irradiation was at most 30 seconds. Local photostimulation experiments were performed by preferentially activating the Mito-KillerRed in the cell body or in TMs.

#### Metabolomic analysis

For ^13^C stable isotope tracer analysis, 6 × 10^6^ GSCs were seeded on transwells for 24h, then treated with siCONT and siFASTKD2.898. After 48 hours, cells were rinsed twice in PBS and 20 ml media (DMEM without glucose, without glutamine, 10% FBS, 1×penicillin–streptomycin) containing 5 mM glucose (unlabelled or [U^13^C6]-D-Glucose, Cambridge Isotope Laboratories, CLM-1396–1) or 2 mM glutamine (unlabelled or [U-^13^C5]-Glutamine, Cambridge Isotope Laboratories, CLM-1822-H-0.1) was added to the cells for 6 h. TM were collected and pelleted and snap frozen, after which the metabolites were extracted into solvent by adding 2 ml of ice-cold chloroform : methanol (2:1 v/v). The suspension was bath sonicated for 3 min, and 2 ml of cold water was added. Then, 1 ml of chloroform: methanol (2:1 v/v) was added to the samples followed by bath sonication for 3 min. The lysates were centrifuged (5,000g for 15 min at 4 °C) and the aqueous phase transferred into a new tube. Samples were frozen until analysis using PEPA^.^, PEPA detects the position of carbon labels in isotopically enriched metabolites and quantifies fractional enrichment through the indirect determination of ^13^C-satellite peaks using one-dimensional ^1^H-NMR spectra.

#### LC–MS analyses

Metabolites were extracted from the frozen cell pellets by adding 200 μl of cold acetonitrile: water (1:1) with 1% metaphosphoric acid and 0.1% formic acid. Samples were vortexed for 30 s and immersed in liquid N_2_ to disrupt cell membranes followed by 30 s of bath sonication; these two steps were repeated three times. Then, samples were incubated at −20 °C for 2 h. Finally, samples were centrifuged at 17,000g for 15 min and the supernatant was collected into a LC–MS vial. Samples were injected in an ultra-high performance liquid chromatography system (1290 Agilent) coupled to a triple quadrupole (QqQ) mass spectrometer (6490 Agilent Technologies) operated in multiple reaction monitoring (MRM) and positive (POS) or negative (NEG) electrospray ionization mode. Metabolites were separated using C18-RP (ACQUITY UPLC BEH C18 1.7 μM, Waters) chromatography at a flow rate of 0.3 ml min^−1^. The solvent system was A (20 mM ammonium acetate,15 mM NH_3_ in water) and B (acetonitrile: water (95:5)). The linear gradient elution started at 100% A (time 0–2 min), 65% A (time 2–5 min) and finished at 100% B (time 5.5 min).

#### TM RNA isolation and RT–qPCR

TMs, cell bodies, and their respective mitochondria were isolated as described above. Six filters containing TMs were combined into one sample. Total RNA of the tubule was isolated using the Direct-zol RNA Miniprep Kit (Zymo Research). Total RNA (1 μg) was used for cDNA synthesis using the High-Capacity cDNA Reverse Transcription Kit (Thermo Fisher Scientific). qPCR was performed using SYBR Green Master mix on the Applied Biosystems 7900HT Real-Time PCR System. The primer sequences are listed in Supplementary Table 6.

#### RNA immunoprecipitation

TMs, cell bodies, and mitochondria were isolated from GSCs as described above, and RNA immunoprecipitation was performed as described in a prior report^86^. Briefly, mitochondria from UV-crosslinked GSCs were extracted in 500 μL extraction buffer (50 mM Tris-HCl [pH 7.5], 150 mM NaCl, 1 mM MgCl2, 100 U/ml RNase inhibitor, 3 mM ribonucleoside vanadyl complex, 1s% NP-40, and complete protease inhibitors without EDTA) on ice for 40 minutes, with occasional vortexing. The extract was centrifuged at 25,000 × g at 4°C for 40 minutes, and the supernatant was pre-cleared overnight with non-coated Dynabeads protein A to reduce non-specific RNA binding. Following washes, primary antibodies were added for overnight incubation. The pre-cleared extract was then equally divided and used for immunoprecipitation with antibody-crosslinked beads and non-coated beads (control immunoprecipitation). The immunoprecipitation reaction was performed at RT for 2 hours.

To isolate mitochondrial RNA following the immunoprecipitation reaction, the beads were washed five times with extraction buffer, then incubated for 30 minutes at 37°C with DNase I, followed by a 30 minutes incubation at 55°C with proteinase K in the presence of 0.1% SDS. Samples were then supplemented with EDTA (5 mM), the magnetic beads were discarded, and phenol-chloroform extraction of RNA was subsequently performed. cDNA was prepared using the High-Capacity cDNA Reverse Transcription Kit (Thermo Fischer Scientific). Quantitative real-time PCR was performed using Applied Biosystems 7900HT cycler.

#### RNA stability assay

GSCs were transfected with FASTKD2 siRNA or siCONT and treated with Actinomycin D (5 μg/mL) for varying timepoints of 0, 3, 6, 9, and 12 hours. Next, TMs and cell bodies were isolated, as described above. For each time point, six filters of tubule isolation were combined into one sample. Isolated RNA was subsequently used for RT-qPCR analysis to examine the expression of specific genes.

#### Chromatin isolation by RNA purification (ChIRP) western blotting

Tumour tubules and mitochondria were extracted as described in the sections above. For ChIRP, biotinylated probes targeting mitochondrial RNA were hybridized with the lysate at 37°C for 4–6 hours, followed by capture with streptavidin-coated magnetic beads. After stringent washes with high-salt buffer, RNA-protein complexes were eluted, and proteins were denatured in SDS-PAGE loading buffer at 95°C for 5–10 minutes. Proteins were separated via SDS-PAGE, transferred to a membrane, probed with primary antibodies, washed, incubated with secondary antibodies for 1 hour at room temperature, and imaged using the Bio-Rad Image System.

#### RNA-seq analysis

RNA-seq analysis was performed on matched cell bodies and TMs isolated from GSCs. Briefly, RNA was extracted and used for library construction using the Illumina TruSeq Stranded Total RNA Library Prep Kit, which was performed by Novogene. The library was sequenced with paired-end 150 bp reads. Raw FASTQ reads were trimmed using Trim Galore, followed by transcript mapping using HISAT2 to the human reference genome (hg38). Samtools was used for sorting, indexing and format conversion from SAM files. Quantification and differential analysis were performed using FeatureCounts and DESeq2 with paired sample analysis. The list of significance was established by setting the fold change threshold at a level of 2 and P < 0.05 using DEseq2. The differentially expressed gene lists generated were subsequently analysed for the enrichment of biological themes using ClusterProfiler or GSEA.

#### Fluorescence in situ hybridization (FISH)

The FISH procedure used in this study has been described in detail previously^86^. The following probe sequence for the 12S MT-RNR1 probe was used: 5′Cy3_TGGCTGGCACGAAATTGACCAACCCTGGGGTTAGTATAGCTTAGTTAAAC-3′ (ITD). Probes were resuspended in 5 μL of formamide, denatured at 75°C for 7 minutes, and added to 35 μL of EXPRESSHyb solution. For endogenous RNA FISH, GSC TMs were fixed for 10 minutes at RT with 4% formaldehyde, 4% sucrose in PBS for 10 minutes, followed by washes with PBS. Cells were permeabilized in ice-cold 70% ethanol for at least 1 hour at 4°C. Cells were then rehydrated in FISH wash buffer (2× SSC, 10% formamide) for 10 minutes. Next, cells were incubated with 50 nM Cy3 labelled probe set in hybridization buffer for 16 hours at 37°C in a humidified chamber. After two washes for 30 minutes at 37°C in 2× FISH wash buffer, coverslips were mounted using antifade mounting agent.

For FISH immunofluorescence, fixed cells were permeabilized with 0.1% triton in PBS for 15 min, followed by 5-minute washes with PBS. Cells were blocked for 30 minutes with 0.5% BSA in PBS before incubating with primary antibody for 1 hour at room temperature. Coverslips were washed for 5 minutes with PBS and incubated for 1 hour with secondary antibody (anti-rabbit Alexa647) at room temperature. Following 5-minute washes in PBS, cells were fixed in 4% paraformaldehyde, and 4% sucrose in PBS for an additional 10 minutes. Following additional washes in PBS, cells were used for FISH as described above.

#### Mass spectrometry analysis of TMs

Microfluidic chambers (Xona Microfluidics) were pre-coated with poly-d-lysine and laminin. 10^5^ cells were cultured in chambers. After 72 hours in the microfluidic devices, lysis solution (50 μL) was flushed through the compartments, and cells were collected and snap-frozen on dry ice. Six compartments of TMs were combined into one sample, added to loading buffer, denatured, and ran on a gel. Gel bands were cut into 1 mm^2^ pieces, de-stained, reduced using DTT, alkylated with iodoacetamide, and subjected to enzymatic digestion with chymotrypsin overnight at 37°C. After digestion, the supernatant was pipetted into a sample vial and loaded onto an autosampler for automated LC-MS/MS analysis. LC-MS/MS experiments were performed using a Dionex Ultimate 3000 RSLC nanoUPLC system and a Q Exactive Orbitrap mass spectrometer. Separation of peptides was performed by reverse-phase chromatography at a flow rate of 300 nL/minute and a Thermo Scientific reverse-phase nano Easy-spray column. Peptides were loaded onto a pre-column (Thermo Scientific PepMap 100 C18, 5 μm particle size, 100A pore size, 300 μm i.d. × 5 mm length) from the Ultimate 3000 autosampler with 0.1% formic acid for 3 minutes at a flow rate of 10 μL/minute. After this period, the column valve was switched to allow elution of peptides from the pre-column onto the analytical column. Solvent A was water + 0.1% formic acid and solvent B was 80% acetonitrile, 20% water + 0.1% formic acid. The linear gradient employed was 2–40% B in 30 minutes. The LC eluant was sprayed into the mass spectrometer using an Easy-Spray source. All m/z values of eluting ions were measured in an Orbitrap mass analyser, set at a resolution of 70000, and scanned between m/z 380–1500. Data-dependent scans (Top 20) were employed to automatically isolate and generate fragment ions by higher energy collisional dissociation (HCD, NCE:25%) in the HCD collision cell, and measurement of the resulting fragment ions was performed in the Orbitrap analyser, set at a resolution of 17500. Singly charged ions and ions with unassigned charge states were excluded from being selected for MS/MS, and a dynamic exclusion window of 20 seconds was employed. For label-free quantification of proteins, peptide identification and relative quantification was carried out in Proteome Discoverer version 2.3. A standard label free quantification workflow was utilized with the Mascot search algorithm. Search parameters included: trypsin as the proteolytic enzyme, with maximum of two missed cleavages, variable oxidation modification of methionine, and deamidation of asparagine and glutamic acid. Fixed carbamidomethylating modification of cysteine and precursor and fragment mass tolerances of 20 ppm and 0.1 Da, respectively, were used. The false discovery rate (FDR) was set at < 1% with two peptide matches to proteins considered reliable. For analysis of SILAC labelled proteins, we used Marquand in addition to Proteome Discoverer. Raw data was processed using Maxquant (version 1.6.1.0) with default settings. MS/MS spectra were searched against the protein sequences. Enzyme specificity was set to trypsin/P, allowing a maximum of two missed cleavages. The minimal peptide length allowed was set to seven amino acids. Global false discovery rates for peptide and protein identification were set to 1%. The match-between runs and re-quantify options were enabled. To avoid false positives, we analysed only RPs where more than two labelled peptides were detected by the software or if the MS spectrum of the detected labelled peptides showed clear peaks at the expected m/z value.

#### Virtual screening docking

The 3D crystal structure of FASTKD2 was obtained via AlphaFold2 (https://alphafold.ebi.ac.uk/). The protein was further refined in Discovery Studio 3.5 by removing all water molecules, adding hydrogen atoms, removing protein polymorphs, and supplementing the structure with non-intact amino acid residues. Ligands were downloaded and prepared by rectifying their bond angles and bond orders and were subsequently minimized using the CHARMm force field. For drug screening, docking site set −29.1187, 2.25847, 24.4153, 14,7701. Lipinski’s rule of five (ROF) and the ADMET (absorption, distribution, metabolism, excretion, and toxicity) processes were initially used. The Lipinski’s five rules are predicted by using the molecular properties in the computational small molecule protocol in Discovery Studio 3.5, including molecular weight (MWT) ≤ 500, hydrogen bonded acceptor ≤ 10, hydrogen-bonded donor ≤ 5, and ClogP ≤ 5 (or MLogP > 4.15). The ADMET descriptors in the molecular property calculation protocol were then used to predict the ADMET properties, where absorption, and blood-brain barrier (BBB) thresholds were set as 1 and 3, respectively. The screened ligands were subjected to docking analysis. The initial screening was performed in the Libdock module of Discovery Studio 3.5. LibDock is a rigid docking method with an algorithm based on molecular dynamics annealing. All molecules from the last step were docked into the site of the RAP binding domain, and individual Libdock analysis were performed. Then, all compounds were ranked according to the Libdock scores, and the top-ranked compounds were selected for precise docking by CDock.

#### Protein expression

The MaxCodonTM Optimization Program (V13) was used to optimize the amino acid sequence of the human FASTKD2 and mutant FASTKD2 (D605A) protein. The FASTKD2 and mutant FASTKD2 (D605A) genes were inserted into pET30a expression vectors, and enzyme digestion and sequencing were used to confirm integration. The expression vector was transformed into BL21 (DE3) competent cells, evenly spread on LB plates (containing 50 μg/mL of kanamycin sulphate), and then placed upside down in a 37°C incubator overnight. A single clone from the transformed plate was selected and inoculated into 4 mL of LB medium (containing 50 μg/mL of kanamycin sulphate). This was grown until the OD600 was 0.5–0.8. Isopropyl β-D-1-thiogalactopyranoside (IPTG, 0.2 mM) was then added to the test tube culture medium and placed in a 15°C chamber for 16 hours to induce expression. The induced culture medium was then centrifuged at 12,000 rpm for 5 minutes. The supernatant was removed, PBS solution was added to resuspend the pellet, SDS-PAGE loading buffer was added to the sample, and the sample was heated at 100°C for 10 minutes and collected via centrifugation. The whole bacteria were further lysed by ultrasonic lysis using 20 mM Tris (pH 8.0), 300 mM NaCl, and 20 mM Imidazole containing 1% Triton. The inclusion bodies were washed with 50 mM Tris (pH 8.0), 300 mM NaCl containing 1% Triton, and 20 mM Imidazole buffer while simultaneously equilibrating the Ni-IDA column. Finally, the target protein was eluted with equilibration buffers of different concentrations of imidazole, and each eluted fraction was collected for SDS-PAGE analysis and detection. After purification and verification analysis by Ni-IDA affinity chromatography, high-purity lanes were collected, added to the treated dialysis bag, dialyzed into buffer (1×PBS [pH of 7.4], 4 mM GSH, 0.4 mM GSSG, 0.4 M L-arginine, 1 M urea], and the renatured protein was finally dialyzed into the storage (1×PBS, pH of 7.4) solution for 6–8 hours. After dialysis and renaturation, the supernatant was filtered with a 0.22 μm filter, aliquoted, and frozen at −80°C.

#### Surface Plasmon Resonance (SPR) assay

SPR measurements were conducted on a Biacore T200 instrument (GE Healthcare) using carboxymethylated dextran (CM) sensor chips pre-immobilized with NTA. The sample compartment was maintained at 15 °C, while the assay temperature was set to 25 °C. Purified protein samples were diluted to 80 μg/ml in HEPES-based immobilization buffer and immobilized onto the sensor surface at a flow rate of 20 μl/min. Residual activated carboxyl groups were quenched by injecting 100 μl of blocking solution (Nicoya). The immobilized protein was then equilibrated with running buffer (PBS, pH 7.4, containing 1% DMSO) until a stable baseline was achieved. Drug solutions prepared in matching running buffer were injected at 20 μl min^–1. Association was monitored for 240 s, followed by a 300 s dissociation phase. Kinetic data were analysed using TraceDrawer software (Ridgeview Instruments).

#### GSC dataset interrogation and analysis

RNA-seq data for GSCs and DGCs were downloaded from GSE54791. And RNA-seq data for GSCs and NSCs were downloaded from GSE41031. RNA-seq-derived counts-per-million matrix was obtained, and differential expression analysis was performed using R package “limma” to obtain a fold change ranking metric for GSC vs. DGC comparison and GSC vs. NSC comparison. To identify the neuron development related pathways that are highly active in GSCs, neuron development related signatures were queried using R package “GSVA” to get the gene signature score. RNA-seq data for 44 GSC and 9 NSC cell lines were downloaded from GSE119834.

#### Public glioma patient database

Public GBM databases from GlioVis (http://gliovis.bioinfo.cnio.es) were used to interrogate FASTKD2 expression levels in GBM and normal tissues and correlation of FASTKD2, SOX2, CD44, AMPA receptors, GABA receptors and AchR receptors. To identify the neuron development related pathways that are highly active in GBM, neuron development related signatures were queried using R package “GSVA” to get the gene signature score. The R package “GSVA” was used to calculate a gene signature score with the “ssgsea” method. For survival analysis, only IDH-wild-type glioma samples were included, and the data were evaluated using Kaplan–Meier survival curves and the log-rank test.

#### Statistical analysis

All analysis was performed using data from at least three independent biological replicates. For all statistical tests, p < 0.05 was considered statistically significant. *, p < 0.05; **, p < 0.01; ***, p < 0.001; ****, p < 0.0001. Statistical tests, including two-tailed unpaired t-tests, one-way ANOVA, two-way ANOVA, Pearson or Spearman correlations, and log-rank tests, were performed using RStudio (R Version 4.5, Boston Massachusetts, USA) or GrapPad Prism 9 (La Jolla, California, USA).

## Supplementary Material

Supplementary Files

This is a list of supplementary files associated with this preprint. Click to download.

• Supplementalfigure7.jpg

• SupplementaryTable5.xlsx

• SupplementaryTable6.xlsx

• Supplementalfigure3.jpg

• SupplementaryTable4.xlsx

• Cy3UTPRNAgranulesinTM.mov

• ExtendedDataFigure.pdf

• ThedynamicsofFASTKD2.mov

• Supplementalfigure1.jpg

• SupplementaryTable1.xlsx

• SupplementaryTable2.xlsx

• Supplementalfigure4.jpg

• Effectoflocalproteinsynthesisinhibitiononintercellularcalciumsignalingingliomacellwithinmicrofluidicplatforms.mov

• Supplementalfigure5.jpg

• SupplementalFigure2.jpg

• InvivocalciumsignalingfollowingFASTKD2knockdown.mov

• SupplementaryTable3.xlsx

• Supplementalfigure6.jpg

• ImpactofFASTKD2onintercellularcalciumwavepropagationinglioma.mov

• Invivocalciumsignalingfollowinglinezolidtreatment.mov
